# Usual Nutrient Intake Distribution and Prevalence of Nutrient Intake Inadequacy among Japanese Children and Adults: A Nationwide Study Based on 8-Day Dietary Records

**DOI:** 10.3390/nu15245113

**Published:** 2023-12-14

**Authors:** Nana Shinozaki, Kentaro Murakami, Shizuko Masayasu, Satoshi Sasaki

**Affiliations:** 1 Department of Nutritional Epidemiology and Behavioural Nutrition, Graduate School of Medicine, The University of Tokyo, 7-3-1 Hongo, Bunkyo-ku, Tokyo 113-0033, Japan; nana-s@m.u-tokyo.ac.jp; 2 Department of Social and Preventive Epidemiology, School of Public Health, The University of Tokyo, 7-3-1 Hongo, Bunkyo-ku, Tokyo 113-0033, Japan; stssasak@m.u-tokyo.ac.jp; 3 Ikurien-Naka, 3799-6 Sugaya, Naka-shi, Ibaraki 311-0105, Japan; sizuko-masa@themis.ocn.ne.jp

**Keywords:** adolescents, adults, children, Dietary Reference Intakes, DRIs, MSM, recommended intakes

## Abstract

In this cross-sectional study, we evaluated nutrient intake adequacy in 4450 Japanese people aged 1–79 years. Dietary data was collected through non-consecutive 8-day weighed dietary records. Usual nutrient intakes from foods and beverages were estimated using the Multiple Source Method. Participant proportions with intakes below and above the Japanese Dietary Reference Intakes (2020) were calculated. Usual intakes of most nutrients were below the Estimated Average Requirement; calcium showed a high percentage of inadequacy across all sex and age groups (29–88%), and iron showed a high inadequacy among females aged 12–64 years (79–95%). The percentages of energy from protein and carbohydrates, dietary fibre, and potassium were typically below the lower limit of the Tentative Dietary Goal for Preventing Lifestyle-related Diseases (DG). Furthermore, over 20% of the participants exceeded the upper limit of the DG for the percentages of energy from total and saturated fats, and over 88% exceeded the upper limit of the DG for sodium. Japanese children and adults could improve their nutrient intake by increasing calcium, iron, dietary fibre, and potassium and reducing total and saturated fats and sodium. These findings can inform policies and interventions to improve nutrient intake in Japan.

## 1. Introduction

A suboptimal diet is a major risk factor for death worldwide [1]. Despite the perception that the Japanese diet is healthier than other diets [2], its overall diet quality is comparable to that of Americans, owing to the high consumption of refined grains and salt and the low intake of dairy products and fruits [3]. In particular, high sodium intake has been identified as a considerable dietary concern in Japan [1,4]. Diet-related health problems are also serious. According to the results of the National Health and Nutrition Examination Survey, the percentage of Japanese males who are overweight or obese has increased in recent years, with one in three males being overweight or obese (body mass index [BMI] ≥ 25 kg/m^2^) [5]. In addition, 21% of females in their 20s are underweight (BMI < 18.5 kg/m^2^), and 12 to 21% of older adults tend to be undernourished (BMI ≤ 20 kg/m^2^) [5]. Furthermore, hypertension is prevalent in 48% of Japanese adults [5]. To address these problems, developing appropriate public nutrition policies is of great importance in Japan.

To develop effective nutrition strategies, it is essential to collect information on the distribution of nutrient intake and the prevalence of nutrient inadequacies in the population [6]. However, due to within-individual variation in dietary intake, utilising dietary data from a single day or averaging dietary data over several days can introduce biases when estimating the proportion of the population with nutrient intakes above or below recommended levels [6,7]. Therefore, nutrient adequacy is generally assessed based on the usual intake distribution estimated using various statistical methodologies, such as the National Cancer Institute method and Multiple Source Method (MSM) [7,8,9]. To date, the distribution and adequacy of usual nutrient intakes have been assessed in nationally representative samples in many countries in the Americas [10,11,12,13,14], Europe [15,16,17,18,19,20,21,22], and Asia [6,7,23,24], based on multiple-day dietary records (DRs) or recalls.

However, little is known about the distribution of usual nutrient intakes in the Japanese population. Although the National Health and Nutrition Survey has been conducted on representative samples of Japanese children and adults [25], the survey only collects one-day dietary data at the household level, which does not allow for an assessment of nutrient intake adequacy. Meanwhile, several individual-level DR surveys have comprehensively assessed the usual nutrient intakes of the Japanese and suggested certain nutrient inadequacies, notably high sodium intakes [26,27,28]. However, the populations in these studies were limited in terms of size (*N* ≤ 910), age groups (3–5 years, 8–14 years, and 20–69 years), and residential areas (≤24 prefectures out of 47). Furthermore, despite the seasonal variation in the Japanese diet [29], none of the studies conducted DRs covering all four seasons. The absence of information on usual nutrient intakes in Japan hampers nutritional assessment and policy making, including the development of Dietary Reference Intakes (DRIs) for the Japanese population [30], which are largely based on the current intakes of the Japanese.

The purpose of this study was to determine the distribution of usual nutrient intakes from food and beverages in a nationwide sample of Japanese children and adults (1–79 years) and assess the adequacy of nutrient intakes. To estimate the usual nutrient intake, we used the MSM to analyse 8-day-weighed DRs, which were collected over a two-day period in each of the four seasons. Drawing from previous studies [26,27,28], we hypothesised that usual intakes are inadequate for several nutrients in both children and adults.

## 2. Materials and Methods

### 2.1. Study Population

This cross-sectional study used data from the MINNADE (MINistry of Health, Labour and Welfare-sponsored NAtionwide study on Dietary intake Evaluation) survey. Details of the survey are available elsewhere [31]. Briefly, the survey consisted of three rounds of one-year data collection (first round: November 2016 to September 2017; second round: October 2017 to September 2018; third round: November 2019 to August 2020). The study included healthy Japanese individuals aged 1–79 years residing in the community. First, we selected 32 geographically diverse prefectures (out of 47) that accounted for more than 85% of Japan’s total population, taking into consideration the feasibility of the survey and the proportion of the population in each region [31,32]. The research dietitians at each prefecture (*N* = 453) were responsible for recruitment and data collection. For the first round of data collection, we included 256 individuals (128 males and 128 females) from each of the nine age bands (1–6, 7–13, 14–19, 20–29, 30–39, 40–49, 50–59, 60–69 and 70–79 years). This totalled 2304 participants. After accounting for the dropout rate from the first round, we recruited 110–119 participants for each sex-age category for the second round, totalling 2051 participants. In the third round, data were further collected from 438 children aged 1–6 years.

The primary inclusion criterion was free-living individuals who could conduct a DR independently or with the help of a guardian for children. The exclusion criteria comprised individuals who were dietitians themselves or lived with dietitians, individuals working with a research dietitian, individuals who had received dietary counselling from a doctor or dietitian, those undergoing insulin or dialysis treatment, pregnant or lactating women, and infants who exclusively consumed human milk. The survey participants were not randomly selected, and only one individual per household was eligible to participate in the survey.

**Figure 1:**
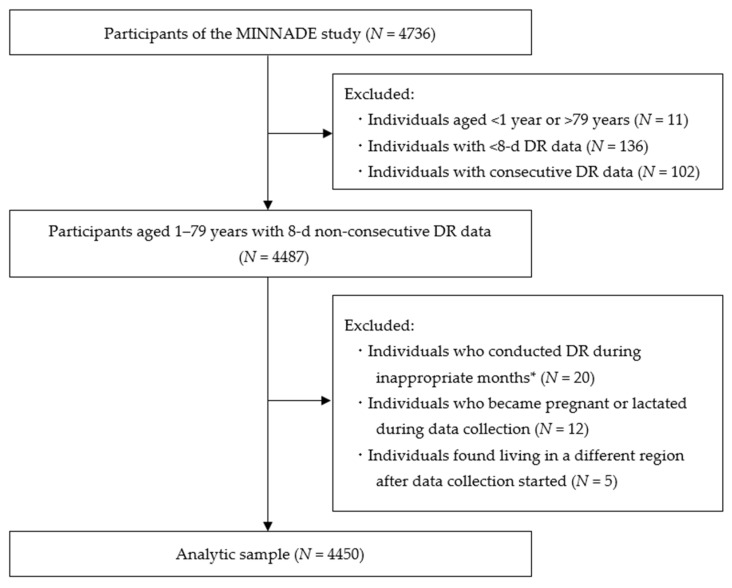
Flow diagram of participant selection in the present analysis. DR, dietary record; MINNADE, MINistry of health, labour and welfare-sponsored NAtionwide study on Dietary intake Evaluation. * Appropriate months were considered October, November, and December for fall; January, February, and March for winter; April, May, and June for spring; and July, August, and September for summer.

Excluding dropouts, 4736 individuals participated in the MINNADE survey (first round: *N* = 2263; second round: *N* = 2036; third round: *N* = 437). All participants were asked to complete two non-consecutive days of the DR during each of the four seasons (8 days in total). Figure 1 illustrates the flowchart of the selection process for participants. We excluded 11 participants aged <1 year or >79 years, 136 who conducted the DR for less than 8 days, 102 with consecutive DR data, 20 who did not complete the DR during the appropriate months (i.e., October, November, and December for fall; January, February, and March for winter; April, May, and June for spring; and July, August, and September for summer), and five who were found living in a different geographic area than the one where they were initially recruited from (identified after data collection). Consequently, the current analysis involved 4450 participants, aged 1’79 years.

### 2.2. Dietary Assessment

Dietary intake information was collected using the DR for two non-consecutive days in each of the four seasons, totalling 8 days. The details of the DR are provided elsewhere [31]. Briefly, each set of two recording days in each season comprised two weekdays (Monday to Friday) for half of the participants and one weekday and one weekend day (Saturday, Sunday, or national holidays) for the other half. This approach was taken to obtain approximate overall dietary data, with a 3:1 ratio (actually 5:2) of weekdays to weekends, while ensuring the feasibility and simplicity of the survey. The research dietitians provided participants with both verbal and written instructions on how to maintain the DR. The participants were requested to weigh and record all foods and beverages they consumed using a digital scale provided. The scale can measure up to 2 kg in 1 g increments (KS-812WT, Tanita, Tokyo, Japan).

The main items recorded were as follows: (i) Dish names, (ii) food names (including ingredients in mixed dishes), and (iii) measured weights or approximate amounts of foods and beverages. The research dietitians collected recording sheets within a few days after each recording day, usually the following day. They then checked the completeness of the DR, adding information if necessary. Each food item was assigned a food code from the Standard Tables of Food Composition in Japan (STFCJ) [33] in a consistent manner. For packaged foods and home-prepared meals, the dietitians estimated the weight of each ingredient as accurately as possible using information on approximate portion sizes, restaurant and manufacturer websites, ingredient labels, nutritional information on food packages, and cookbooks. Other research dietitians later reconfirmed all food codes and weights at the central office of the study. Finally, the STFCJ [33] was used to calculate daily energy and nutrient intakes from foods and beverages, excluding dietary supplements, for each participant.

### 2.3. Assessment of Basic Characteristics

At the beginning of each survey round, participants were measured barefoot and in light indoor clothing by family or research dietitians using standardised procedures to obtain body height (to the nearest 0.1 cm) and weight (to the nearest 0.1 kg). For participants who could not be measured for weight or height (*N* = 92), self-reported information was used. BMI was calculated as body weight (kg) divided by the square of height (m^2^). A questionnaire was used to gather information about the basic characteristics of the participants. Age (years) at the beginning of the study was calculated from the date of birth. Residential area was grouped into 1 of 6 regions (Hokkaido and Tohoku, Kanto, Hokuriku and Tokai, Kinki and Chugoku, or Kyushu and Okinawa). Annual household income was classified into three categories based on distribution: <5, ≥5 to <8, and ≥8 million Japanese yen. Educational attainment was assessed among adults and classified as follows: Junior high or high school, junior college or technical school, university or higher, or other. The employment status of adult participants was categorised into four categories: Student, unemployed, part-time, or full-time. The smoking habits of adults were categorised into three types: current smoker, past smoker, or non-smoker.

### 2.4. Handling of Dietary Misreporting

Misreporting of energy intake (EI) was evaluated based on the ratio of EI to estimated energy requirement (EER) [34]. EER was calculated using equations published in the DRIs of the US/Canada, based on sex, age, body height and weight, and physical activity [35]. Owing to the absence of an empirical measure of physical activity, we adopted ‘low-active’ for physical activity level [35]. Under-reporters, plausible reporters, and over-reporters were identified based on the cut-off values of the EI:EER calculated from the number of recording days (8 days), within-subject variation in EI at 23%, day-to-day variation in total energy expenditure at 8.2%, and the coefficient of variation in intakes and other components of energy balance (i.e., error in the EER equations at 2.97–14.8% according to sex and age) [34,36]. However, misreporters were not excluded from the analysis, as exclusion would have introduced bias [37,38].

### 2.5. Estimation of Usual Intakes

The usual intake distribution of energy and nutrients was estimated using the MSM [39]. Briefly, the MSM estimates the consumption probability of a nutrient using logistic regression and then applies linear regression to compute the usual consumption-day amount for each individual. Finally, the usual intake on all days is computed by multiplying consumption probability and consumption-day amount [39]. We entered 8-day measurements of energy and nutrient intakes for each participant, including the percentages of energy from protein, total fat, saturated fats, and carbohydrates into the MSM online interface (https://msm.dife.de/, accessed on 23 October 2023). In this study, the consumption probability was set to one, assuming that all participants were habitual consumers of all nutrients evaluated. Covariates in the usual intake models included age (years), sex (male or female), and day of the week (weekday or weekend day) [8]. We also estimated the usual intake without covariates; however, this did not affect the subsequent assessment of nutrient inadequacy.

From the results of the MSM program, we obtained the usual intake of energy and each nutrient for each participant. Additionally, we obtained the usual intake distribution at the group level by sex, including the mean, standard deviation (SD), minimum and maximum values, and percentile distribution, for each of the 12 age groups adopted in the Japanese DRIs (i.e., 1–2 years, 3–5 years, 6–7 years, 8–9 years, 10–11 years, 12–14 years, 15–17 years, 18–29 years, 30–49 years, 50–64 years, 65–74 years, and ≥75 years) [30].

### 2.6. Data Analysis

The adequacy of nutrient intake was assessed by comparing usual nutrient intakes with age- and sex-specific reference intakes in the Japanese DRIs [30]. The Japanese DRIs consist of several nutrient indicators with different objectives. The Estimated Average Requirement (EAR) is “the amount that would meet the nutrient requirements of 50% of the population” [30]. The Adequate Intake (AI), defined as “the amount that is adequate to maintain a certain nutritional status”, is used when the EAR cannot be determined in order to evaluate the median intake of a group [30]. The dietary intake of no less than the AI minimises the risk of inadequacy [30]. The Tolerable Upper Intake Level (UL) is “the maximum amount that avoids adverse health effects due to excessive intake”. The tentative dietary goal for preventing lifestyle-related diseases (DG) is the amount of nutrients necessary to prevent lifestyle-related diseases. It specifies the recommended intake ranges for macronutrient balance (the percentages of energy from protein, total fat, saturated fats, and carbohydrates), dietary fibre, sodium, and potassium. These indicators for nutrients were determined assuming physical activity level II (normal).

Of the 33 nutrients presented in the Japanese DRI, we have excluded five nutrients (i.e., biotin, iodine, selenium, chromium, and molybdenum) from the present analysis as the data for these nutrients in the STFCJ were insufficient [33]. For the remaining 28 nutrients, the proportion of inadequate intake was assessed based on the percentage of participants who did not meet the DRIs using the cut-point method (except for iron, as described later) [40]. The reference values for energy and nutrients are shown in Supplementary Tables S1–S4. For the EAR, an intake level below the EAR was considered inadequate for the following nutrients: Protein, vitamin A (retinol equivalents), thiamine, riboflavin, niacin (niacin equivalent), vitamin B-6, vitamin B-12, folate, vitamin C, sodium, calcium, magnesium, zinc, and copper [30,41]. For iron, the cut-point method is not suitable for children and menstruating females because their iron requirements are not normally distributed [40,42]. Therefore, the iron intake deficiency of these populations was assessed using the full probability method based on the values published by the World Health Organization (WHO) [42]. First, assuming the bioavailability of iron from the diet is 15% [30], the amount of iron that would result in a 50% chance of iron deficiency was determined (i.e., less than 3.6, 4.9, and 9.3 mg/day for children aged 1–2 years, children aged 3–9 years, and females aged 12–64 years, respectively) [42]. Thus, inadequate iron intake was identified when the individual’s iron intake was below these levels.

For nutrients with the DG (percentage of energy from protein, total fat, saturated fat, carbohydrates, dietary fibre, sodium, and potassium), we calculated the percentage of participants consuming nutrient intake outside (below or above) the range of the DG [30]. We also computed the percentage of participants whose intake was above the UL for 11 nutrients (i.e., vitamins A, D, E, and B-6, niacin, calcium, phosphorus, iron, zinc, copper, and manganese). The UL for niacin is presented as the amount of nicotinamide or nicotinic acid in the DRIs. Therefore, the adequacy of niacin intake was assessed by comparing the usual niacin intake with the sum of nicotinamide and nicotinic acid. The AI is set for nine nutrients in the DRIs (*n*-6 and *n*-3 polyunsaturated fatty acids, vitamins D, E, and K, pantothenic acid, potassium, phosphorus, and manganese). Although a person’s usual intake could be below the AI, it cannot be concluded that the intake is inadequate since the person’s actual requirement could be considerably lower than the AI values [43]. Nevertheless, if a group mean or median intake is below the AI, it may be desirable for intakes to increase [40,44]. Accordingly, we identified if the group’s median intake was below AI or not for the nine nutrients [30]. Analyses were conducted using the statistical software package SAS version 9.4 (SAS Institute Inc., Cary, NC, USA).

## 3. Results

### 3.1. Basic Characteristics of the Participants

This analysis included 1648 children and adolescents aged 1–17 years and 2802 adults aged 18–79 years (Table 1). The mean BMI was 17.2 kg/m^2^ for children and adolescents and 23.0 kg/m^2^ for adults. The 8-day mean EI was 1766 kcal/day (SD: 635) for children and adolescents and 2003 kcal/day (SD: 461) for adults. The mean EI:EER was close to the expected ratio of 1.00 in both children and adults, ranging from 0.90 to 1.14. Of the participants, 14% were under-reporters, 78% were plausible reporters, and 9% were over-reporters. Among the under-reporters, 53% were children aged 1–5 years, while among the over-reporters, 78% were adults aged 18–49 years.

### 3.2. Adequacy of Nutrient Intakes in Children and Adolescents

Table 2 and Table 3 present the mean usual intakes and the proportion of children and adolescents who did not meet the DRIs for energy and nutrients in males and females, respectively. In both sexes, almost all children and adolescents had intakes above the EAR for protein, niacin, vitamin B-12, and copper. However, a high percentage of children and adolescents had intakes below the EAR for other nutrients. For example, the estimated prevalence of inadequacy was high for vitamin A (3–57%), thiamine (7–52%), riboflavin (3–62%), vitamin C (5–53%), and calcium (29–83%). In addition, more than 91% of females aged 12–17 years had an insufficient iron intake, as assessed by the probability method. The intake of vitamin B-6, folate, magnesium, and zinc was generally adequate for children under 12 years, but mostly inadequate for those aged 12–17 years.

The DG was not in many nutrients. For instance, a high proportion of children and adolescents had intakes below the lower limit of the DG for the percentages of energy from protein and carbohydrates, dietary fibre, and potassium. Moreover, more than 20% of children and adolescents in each sex and age category had intakes exceeding the upper limit of the DG for the percentages of energy from total and saturated fats. Furthermore, almost all children (≥95%) exceeded the upper limit of the DG for sodium.

The AI was met in all sex and age groups for *n*-6 polyunsaturated fatty acids, vitamins E and K, and potassium (i.e., the group median usual intake was equal to or above the AI). However, the median usual intake of vitamin D and manganese was below the AI for both sexes in the 10–17 age group. None of the participants exceeded the UL for all nutrients, except for vitamin A. The percentage of participants exceeding the UL for vitamin A ranged from 0.5% to 4.9% for children aged 1–5 years. The distributions, including the 5th to 95th percentiles of energy and nutrient intakes among children and adolescents, are shown in Supplementary Tables S5–S11.

### 3.3. Adequacy of Nutrient Intakes in Adults

Table 4 and Table 5 show the nutrient intakes of male and female adults, respectively. In both sexes, the proportion of adults with intakes below the EAR was zero or very low for protein, niacin, vitamin B-12, sodium, and copper. However, a high percentage of adults had intakes below the EAR for other nutrients. In particular, more than 5% of male and female adults of all age groups did not meet the EAR for vitamins A, B-6, and C, thiamine, riboflavin, calcium, magnesium, and zinc. Additionally, over 78% of females aged 18–64 years had inadequate iron intake. Inadequate nutrient intakes were more prominent in younger age groups than in older age groups.

Similar to the results for children and adolescents, the DG was not in many nutrients. For instance, a high proportion of adults had intakes below the lower limit of the DG for the percentages of energy from protein and carbohydrates, dietary fibre, and potassium. Moreover, the highest proportion of inadequate protein intake was observed in the 75–79 years age group for both sexes. In addition, over 20% of adults had intakes above the upper limit of the DG for total and saturated fats, and over 88% of adults had sodium intakes above the upper limit of the DG.

The median usual intake was equal to or above the AI for *n*-6 polyunsaturated fatty acids, vitamins E and K, and phosphorus in all sex and age groups. However, the median usual intake of *n*-3 polyunsaturated fatty acids, vitamin D, potassium, and manganese was below the AI for people aged 18–29 years, regardless of sex. Equally, both males and females aged 30–49 had a median usual intake below the AI for vitamin D and manganese, while those aged 50–64 had a median vitamin D intake below the AI. None of the participants exceeded the UL for all nutrients, except for manganese. The percentage of the participants exceeding the UL for manganese ranged from 0.2% to 1.0% for males aged 18–49 and 65–79 years and from 0.2% to 0.8% for females aged 18–64 years. The usual intake distributions of energy and nutrients among adults are shown in Supplementary Tables S12–S16.

## 4. Discussion

### 4.1. Key Findings

To our knowledge, this is the first study to estimate usual nutrient intakes in a large sample of the Japanese population and evaluate the adequacy of these nutrient intakes. Our results showed that the estimated usual intakes in this population were inadequate for most nutrients. For instance, all sex and age groups had a high percentage of inadequate intakes for macronutrients (including dietary fibre, the percentages of energy from protein, carbohydrates, and total and saturated fats) and micronutrients (including vitamins A and C, thiamine, riboflavin, calcium, iron, sodium, and potassium). Of particular concern, 29–88% of the participants had an inadequate calcium intake, and over 88% had an excessive sodium intake. Moreover, more than 20% of the participants had intakes above the recommended upper levels for the percentages of energy from total and saturated fats. Inadequate nutrient intakes were particularly pronounced in adolescents and young adults. The AI and UL were met for most age groups in both sexes with a few exceptions, such as excessive vitamin A intake for children aged 1–5 years. The present findings should serve as a reference for conducting future research and shaping nutrition policy related to nutrient intake and adequacy in Japanese children and adults.

### 4.2. Macronutrient Inadequacy

Macronutrient inadequacy has been documented in Japan [26,27,28] and in many other countries [7,15,18,19,21,24]. Our results similarly indicated that the percentage of energy intake from energy-providing nutrients (i.e., protein, carbohydrates, and total and saturated fats) was inadequate for most age groups in both sexes. In comparison to the Japanese DRI [30], the percentage of energy was generally higher for total and saturated fats and lower for protein and carbohydrates in this study. However, the DG of saturated fats in the Japanese DRI was established in accordance with the median intake of the Japanese population [30], and its recommended intakes for children aged 15–17 years and adults (≤8% and ≤7% of energy, respectively) are more stringent than those of the WHO guideline (i.e., ≤10% of energy intake) [45]. In this study population, the median values for the percentage of energy from saturated fats were not extremely high, ranging from 7% to 10% across sex and age categories. Considering that the effects of saturated fat on health are still inconclusive [46,47], it is debatable whether stricter limits on saturated fat intake should be recommended for the Japanese. By contrast, inadequate protein intake (as a percentage of energy) was observed in males and females of all age groups but was more pronounced in older adults aged 75–79 years. This is a matter of great concern, as insufficient protein intake is a major contributor to age-related muscle loss [48]. Thus, increasing energy intake from protein would be beneficial for the Japanese population, especially older adults.

Insufficient dietary fibre intake has been reported worldwide [1,12,15,19,26,27,49]. Our investigation found that adolescents and adults did not consume enough dietary fibre. Nevertheless, this study saw a lower proportion of inadequacy compared with previous studies in Japan [26,27,28]. For instance, in 2014, the proportion of children aged 8–14 years with dietary fibre intake below the DG was 47% for boys and 43% for girls [28], whereas in this study it was 0% for boys and 2% for girls. This could be ascribed to the revision of the STFCJ rather than an actual increase in dietary fibre intake. The previous studies used the earlier editions of the STFCJ [50,51], which estimated the dietary fibre content of foods using the modified Prosky method. In contrast, this study used the latest edition [33], which uses both the modified Prosky method and the Association of Official Analytical Chemists (AOAC) 2011.25 method. A previous study has suggested that using the latest STFCJ edition would increase total dietary fibre intake, predominantly due to the contribution of rice [52]. Therefore, future dietary surveys should carefully monitor the dietary fibre intake of the Japanese population.

### 4.3. Micronutrient Inadequacy

We observed that a considerable proportion of all sexes and ages had inadequate usual intakes of micronutrients (i.e., vitamins A and C, thiamine, riboflavin, calcium, iron, sodium, and potassium). Inadequate intakes of several vitamins and minerals have also been reported for children and adults in many countries [6,7,10,11,13,14,15,17,18,20,23,24,26,27,28]. For example, calcium intake is far below the optimal levels worldwide [1], including in Japan [26,27,28], China [6,23], the US [10,11], Canada [14], the Netherlands [17], Greece [20], the Philippines [24], and South Korea [7]. Results from this study indicated that 29–88% of the participants, irrespective of their sex or age, had insufficient calcium intake. This may be due to the low consumption of calcium-rich dairy products, such as milk, among the Japanese [1]. Milk is the top contributor to calcium intake in both the US [53] and Japan [5], whereas the average milk consumption of Japanese adults [5] is less than half that of US adults [54]. Moreover, we also found that over 79% of females aged 12–64 years had inadequate iron intake, which was much higher than the rest of the population. Correspondingly, a previous study revealed that 60% of Japanese females aged 13–14 years had inadequate iron intake [28]. The inadequate iron intake may be due to a higher iron requirement in this population. In addition, although fortified cereal foods largely contribute to iron intake in other developed countries [55], it is less common in Japan. Iron deficiency among females of childbearing age is also a serious concern in other countries, including China [6] and the Philippines [24]. Addressing iron deficiency in females of reproductive age is imperative, as iron deficiency anaemia at this stage can have serious maternal and foetal consequences [56].

Another problem is the excessive intake of sodium in both sexes. In this study, 89–100% of participants in each age group had a usual intake above the DG for sodium (as salt equivalent). The DG for sodium was determined as a midpoint between the WHO recommendation (5 g/day) [57] and the median sodium intake of the Japanese population [30]. The high proportion of the excessive sodium intake is, therefore, an expected consequence and indicates that the sodium intake of the Japanese population is much higher than the target level. In fact, our results showed that the mean salt intake among adults ranged from 10.5 g/day to 11.8 g/day for males and from 8.5 g/day to 10.5 g/day for females, almost double the WHO recommendation [57]. A previous study has shown that seasonings, such as salt or soya sauce, and fish and shellfish were the main contributors to sodium intake among Japanese adults [58]. Excessive sodium (salt) intake is a major nutrient problem not only in Japan [26,27,28], but also in China [6], South Korea [7], Finland [18], Greece [20], and the US [12]. Furthermore, our results showed that a high percentage (7–88%) of males and females in all age groups aged 3 years or older had potassium intake below the lower limit of the DG. Given that high sodium intake is the leading dietary risk for death and disability-adjusted life years in Japan [1] and that a high sodium-potassium intake ratio is a risk factor for stroke [59] and all-cause mortality [60], urgent action is needed to reduce sodium intake and the sodium-potassium intake ratio in the Japanese population.

Consistent with previous studies [7,26], the UL was met in most age groups. However, a small percentage (<5%) of participants in some subgroups exceeded the UL for vitamin A in children and manganese in adults. The prevalence of excessive intake of vitamin A by infants and toddlers from food and beverages in this study was comparable to that in the Netherlands (<5%) [17], but lower than that in the US (≥16%) [12]. In Japan, the main dietary sources of retinol are fish and eggs, β-carotene is obtained from carrots and green leaves [61], and manganese is sourced from white rice and Japanese tea [62]. Acute and chronic excessive vitamin A intake leads to liver damage and fibrosis [63]. In addition, the accumulation of manganese in the brain may lead to neurotoxicity and subsequent neurodegenerative diseases [64]. Since dietary supplements were not included in this study, the actual proportion of excess intake may be higher. Therefore, excessive intake of these nutrients from the total diet, including supplements, should be carefully monitored.

Previous studies have shown that nutrient adequacy may be associated with participant characteristics [17,24]. For instance, in the Netherlands, adolescents were more likely to have intakes below the EAR, and older adults were more likely to have adequate intakes [17]. Conversely, older adults in the Philippines and South Korea faced a greater prevalence of inadequacy than younger adults [7,24]. In this study, as the purpose of this study was to provide descriptive information on usual nutrient distribution and nutrient adequacy, no statistical comparisons were made between subgroups, such as by age, sex, and income. However, the intakes of vitamin B-6, folate, magnesium, and zinc were mostly inadequate for boys and girls aged 12–17 years. Moreover, among adults, the proportion of inadequate intakes seemed to be relatively higher in younger age groups than in older age groups. Thus, nutrient inadequacy may be particularly prevalent among adolescents and young adults. Socioeconomic and behavioural correlates of nutrient inadequacy should be explored in future studies.

### 4.4. Social Implications

This study highlighted the low adherence to nutrient intake goals among Japanese individuals. Given the serious health implications of inadequate nutrient intake, such as chronic disease mortality and morbidity [1], there is an urgent need for nutritional strategies to assist the Japanese in meeting nutrient recommendations. For instance, future strategies may include education on healthy food choices, restrictions on food marketing, food supply improvement through reformulation and fortification (e.g., reducing fat and salt and increasing dietary fibre), and dietary supplementation of nutrients that are unlikely to be provided by food intake alone [1,15,22]. However, implementing these strategies requires cross-sectoral policies and interactions between public health organisations [15]. In addition, continuous monitoring of nutrient intakes and health-related indicators is needed to ensure the effectiveness of the strategies.

Nevertheless, given that many values in the Japanese DRIs were derived from dietary guidelines and studies conducted in Western populations [30], these reference values may not be entirely suitable for the Japanese population. Consequently, the estimated proportion of inadequate nutrient intake in this study may differ from the actual percentage. Further studies involving Japanese populations are needed to determine more appropriate reference values.

### 4.5. Strengths and Limitations

The strength of this study lies in the detailed dietary data collected from 8-day DRs for each of the four seasons from a large sample of Japanese children and adults from geographically diverse regions throughout Japan. This survey design enabled us to represent dietary intake by accounting for day-to-day and seasonal variations [29]. To our knowledge, a detailed DR survey on this population size has never been conducted in Japan. Therefore, this study provides an invaluable resource for understanding the dietary intake of the Japanese. However, this study has several limitations. First, our participants were volunteers and not a representative sample of the general Japanese population. Therefore, they may be more health conscious and have healthier diets than the general population. In comparison with the nationally representative sample, the adults in this study had a slightly higher education level [31]. However, the distributions of household income, height, and weight were similar for both children [5,65] and adults [31]. Thus, there is no strong evidence to suggest that the participants in this study significantly differed from the general Japanese population. Second, self-reported dietary assessment methods are subject to both random and systematic measurement errors [37]. In particular, DRs are generally prone to measurement error due to incorrect recording and changes in dietary behaviour [37]. To minimise the possibility of systematic error, research dietitians checked the dietary information recorded by the participants’ caregivers. Our results showed that approximately 80% of the participants were plausible reporters, and the mean EI:EER ratio was close to the expected value, suggesting that dietary reporting bias was minimised. Third, due to the unavailability of composition databases in Japan, we could not account for nutrient intakes from dietary supplements or fortified foods. A recent study reported that 23% of Japanese adults were users of fortified foods or dietary supplements [66]. Subsequently, the proportions of participants with intakes below the recommended levels have likely been overestimated, while those with excessive nutrient intakes may have been underestimated [10,17]. Future studies should consider total nutrient intake, including dietary supplements and fortified foods. Fourth, since this study did not take into account the loss of nutrients during cooking [67], the true proportion of the participants with vitamin intakes below the EAR may be higher than our results. Fifth, the number of participants in each sex and age group ranged from 63 to 443 (mean: 185), with particularly low numbers in children, adolescents, and older adults. Therefore, caution is needed when interpreting the distribution of nutrient intakes in this population. Nevertheless, we analysed the data stratified by sex and age categories of the Japanese DRI [30] in order to provide basic information for its future development. Sixth, we did not include certain populations, such as infants under 1 year of age, individuals over 79 years of age, and pregnant or lactating women. Therefore, future research should include these groups to assess nutrient adequacy and establish nutritional policies. Finally, a general limitation in the assessment of nutrient intakes is that the food composition tables may not necessarily reflect the true nutrient composition of foods, as they may vary according to season and variety [29].

## 5. Conclusions

To our knowledge, this is the first study to present the distribution of usual nutrient intakes and to assess the adequacy of nutrient intakes using detailed DR data in a large sample of the Japanese population. The nutrient intakes of this study population were generally inadequate compared with the Japanese DRIs. Of particular concern were remarkably high intakes of sodium and low intakes of vitamins A and C, thiamine, riboflavin, potassium, calcium, iron, magnesium, dietary fibre, and protein. The results of this study may provide a scientific basis for the development of national dietary guidelines and the design of dietary interventions to improve nutrient intakes in Japan. However, since the study population is not a representative sample of the Japanese population, it is crucial to conduct a national dietary survey utilising detailed dietary assessment methods at the individual level to continually monitor the usual dietary intake of Japanese people.

## Figures and Tables

**Table 1:** Basic characteristics of the study participants (*N* = 4450) ^a^.

Variable	Children and Adolescents (1–17 Years)		Adults (18–79 Years)	
	Males (*N* = 841)	Females (*N* = 807)	Males (*N* = 1375)	Females (*N* = 1427)
Age (year)	7.7 ± 5.0	7.5 ± 4.9	48.4 ± 17.7	48.2 ± 17.7
Body height (cm)	124.3 ± 0.3	120.2 ± 27.3	169.3 ± 6.3	156.3 ± 6.0
Body weight (kg)	29.6 ± 18.2	26.9 ± 14.8	67.9 ± 11.0	54.5 ± 9.1
Body mass index (kg/m^2^)	17.2 ± 2.8	17.1 ± 2.6	23.6 ± 3.4	22.3 ± 3.5
Residential area, *N* (%)				
Hokkaido and Tohoku	93 (11.1)	82 (10.2)	153 (11.1)	163 (11.4)
Kanto	287 (34.1)	288 (35.7)	480 (34.9)	488 (34.2)
Hokuriku and Tokai	144 (17.1)	132 (16.4)	241 (17.5)	252 (17.7)
Kinki and Chugoku	219 (26.0)	201 (24.9)	327 (23.8)	353 (24.7)
Kyushu and Okinawa	98 (11.7)	104 (12.9)	174 (12.7)	171 (12.0)
Annual household income, *N* (%)				
<5 million Japanese yen	118 (14.0)	107 (13.3)	635 (46.2)	531 (37.2)
≥5 to <8 million Japanese yen	355 (42.2)	345 (42.8)	412 (30.0)	447 (31.3)
≥8 million Japanese yen	360 (42.8)	347 (43.0)	317 (23.1)	427 (29.9)
Missing	8 (1.0)	8 (1.0)	11 (0.8)	22 (1.5)
Educational level, *N* (%)				
Junior high or high school	-	-	531 (38.6)	588 (41.2)
Junior college or technical school	-	-	267 (19.4)	558 (39.1)
University or higher	-	-	564 (41.0)	270 (18.9)
Other	-	-	7 (0.5)	9 (0.6)
Missing	-	-	6 (0.4)	2 (0.1)
Employment status, *N* (%)				
Students	-	-	228 (16.6)	292 (20.5)
Unemployed	-	-	56 (4.1)	74 (5.2)
Part-time job	-	-	114 (8.3)	229 (16.0)
Full-time job	-	-	972 (70.7)	832 (58.3)
Missing			5 (0.4)	0 (0)
Smoking status, *N* (%)				
Current smoker	-	-	336 (24.4)	118 (8.3)
Past smoker	-	-	470 (34.2)	148 (10.4)
Non-smoker	-	-	564 (41.0)	1161 (81.4)
Missing	-	-	5 (0.4)	0 (0)
EI ^b^ (kJ)	1930 ± 748	1595 ± 429	2211 ± 479	1803 ± 337
EER, kcal/d	1723 ± 652	1472 ± 460	2479 ± 300	1930 ± 190
EI:EER ^b^	1.14 ± 0.21	1.12 ± 0.20	0.90 ± 0.22	0.94 ± 0.21

EER, estimated energy requirement; EI, energy intake. ^a^ Values are expressed as mean ± standard deviation, unless otherwise indicated. ^b^ EI calculated as an 8-day mean per participant was used.

**Table 2:** Mean (SD) and inadequacy of nutrient intakes from foods and beverages among Japanese male children and adolescents aged 1–17 years (*N* = 841) ^a^.

Variables	1–2 Years				3–5 Years				6–7 Years				8–9 Years				10–11 Years				12–14 Years				15–17 Years			
	(*N* = 149)				(*N* = 225)				(*N* = 98)				(*N* = 63)				(*N* = 77)					(*N* = 107)				(*N* = 122)		
	Mean	SD	Prevalence of Inadequacy (%) ^b^		Mean	SD	Prevalence of Inadequacy (%) ^b^		Mean	SD	Prevalence of Inadequacy (%) ^b^		Mean	SD	Prevalence of Inadequacy (%) ^b^		Mean	SD	Prevalence of Inadequacy (%) ^b^		Mean	SD	Prevalence of Inadequacy (%) ^b^		Mean	SD	Prevalence of Inadequacy (%) ^b^	
**Energy, kcal/d**	1215	203	-		1475	202	-		1715	172	-		1990	303.2	-		2123	314	-		2732	535	-		2960	723	-	
**Nutrients without DRI values**																												
Fat, g/d	38.7	9.9	-		50.1	8.3	-		59.2	9.0	-		70.3	13.8	-		75.4	13.8	-		93.8	20.3	-		100.7	25.4	-	
SFA, g/d	12.6	3.5	-		16.1	3.2	-		19.3	3.1	-		22.6	4.2	-		23.8	4.8	-		29.5	6.7	-		30.6	8.0	-	
Carbohydrate, g/d	174.2	26.4	-		202.9	27.8	-		233.7	26.5	-		266.9	38.1	-		281.7	38.2	-		375.7	79.1	-		407.4	112.4	-	
**Nutrients with EAR**			**<EAR**				**<EAR**				**<EAR**				**<EAR**				**<EAR**				**<EAR**				**<EAR**	
Protein, g/d	42.0	8.8	0		52.5	8.8	0		61.0	7.5	0		71.4	12.5	0		78.5	14.4	0		95.2	18.2	0		104.9	27.1	0	
Vitamin A, μg RAE/d	374	119	25.5		418	119	27.6		433	94	10.2		513	114	3.2		582	186	27.3		645	164	27.1		651	230	50.0	
Thiamine, mg/d	0.60	0.10	6.7		0.70	0.10	10.2		0.90	0.10	7.1		1.00	0.20	11.1		1.10	0.20	24.7		1.40	0.30	28.0		1.50	0.40	30.3	
Riboflavin, mg/d	0.80	0.20	8.1		1.00	0.20	7.6		1.10	0.20	5.1		1.30	0.20	3.2		1.40	0.40	24.7		1.60	0.40	16.8		1.70	0.50	32.0	
Niacin, mg NE/d	16.9	3.9	0		21.2	3.6	0		25.1	4.0	0		29.5	6.0	0		32.7	6.2	0		39.5	8.3	0		44.2	12.3	0	
Vitamin B-6, mg/d	0.80	0.20	1.3		0.90	0.20	0		1.00	0.20	4.1		1.20	0.20	0		1.30	0.30	9.1		1.60	0.40	12.1		1.70	0.50	13.9	
Vitamin B-12, μg/d	3.0	0.9	0		3.7	1.1	0		4.4	1.1	0		5.5	1.7	0		6.0	2.3	0		6.1	1.6	0		6.7	2.5	0.8	
Folate, μg/d	173	45	1.3		203	51	0		220	44	0		262	38	0		285	78	0		330	93	0.9		365	113	9.0	
Vitamin C, mg/d	62	19	6.0		72	23	4.9		73	21	12.2		81	15	7.9		92	32	24.7		109	40	31.8		112	44	24.6	
Sodium, mg	2077	435	-		2582	537	-		3065	541	-		3639	578	-		3958	665	-		4621	860	-		4977	1293	-	
Calcium, mg/d	408	114	31.5		490	149	57.3		531	127	44.9		626	118	28.6		657	210	48.1		729	209	71.0		648	251	58.2	
Magnesium, mg/d	149	29	0		176	34	0		196	33	0		229	34	0		250	52	1.3		297	72	26.2		304	83	48.4	
Iron, mg/d	4.1	1.0	8.1	^c^	4.9	1.0	18.2	^c^	5.6	1.1	28.6	^c^	6.6	1.2	28.6	^c^	7.2	1.5	51.9	^c^	8.6	1.9	35.5	^c^	9.3	2.5	31.1	^c^
	-	-	29.5	^d^	-	-	50.2	^d^	-	-	23.5	^d^	-	-	6.3	^d^	-	-	-		-	-	-		-	-	-	
Zinc, mg/d	5.2	1.1	1.3		6.4	1.1	0		7.5	1.0	0		8.8	1.4	0		9.7	1.9	1.3		12.0	2.4	7.5		13.1	3.4	18.0	
Copper, mg/d	0.60	0.10	0		0.80	0.10	0		0.90	0.10	0		1.00	0.10	0		1.10	0.20	0		1.40	0.30	0		1.50	0.40	0	
**Nutrients with DG**			**<DG**	**>DG**			**<DG**	**>DG**			**<DG**	**>DG**			**<DG**	**>DG**			**<DG**	**>DG**			**<DG**	**>DG**			**<DG**	**>DG**
Protein, % energy	13.8	1.4	21.5	0	14.3	1.0	10.7	0	14.3	1.2	14.3	0	14.5	1.3	14.3	0	14.8	1.1	3.9	0	14.1	1.1	16.8	0	14.2	1.3	21.3	0.8
Fat, % energy	28.0	3.7	2.0	26.8	30.2	2.4	0	55.6	30.8	3.1	0	63.3	31.4	2.2	0	77.8	31.6	2.1	0	80.5	30.5	2.6	0	57.0	30.4	3.6	0	54.1
SFA, % energy	9.1	1.6	-	-	9.7	1.3	-	42.2	10.0	1.4	-	52.0	10.2	0.7	-	65.1	10.0	1.0	-	46.8	9.6	1.0	-	41.1	9.2	1.4	-	81.1
Carbohydrate, % energy	58.0	4.5	5.4	6.0	55.4	2.7	2.2	0	54.8	3.4	2.0	0	53.9	2.8	7.9	0	53.5	2.9	9.1	0	55.2	3.3	8.4	0	55.2	4.4	13.1	1.6
Dietary fibre, g/d	11.1	2.5	-	-	13.4	2.6	0	-	15.3	2.5	0	-	18.0	2.5	0	-	19.2	3.2	1.3	-	24.0	5.5	9.3	-	25.1	6.6	16.4	-
Sodium, g salt equivalent/d	5.3	1.1	-	96.6	6.6	1.4	-	99.1	7.8	1.4	-	100	9.2	1.5	-	100	10.1	1.7	-	100	11.7	2.2	-	100	12.6	3.3	-	95.1
Potassium, mg/d	1563	327	-	0	1839	361	7.1	0	2005	324	27.6	0	2337	331	17.5	0	2541	604	24.7	0	2983	739	21.5	0	3012	839	47.5	0
**Nutrients with AI**																												
*n*-6 PUFA, g/d	5.50	1.40	-		7.20	1.40	-		8.40	1.50	-		10.00	2.50	-		10.90	2.20	-		13.50	3.00	-		14.40	3.80	-	
*n*-3 PUFA, g/d	0.90	0.30	-		1.30	0.30	-		1.40	0.40	-	^e^	1.70	0.50	-		1.90	0.50	-		2.10	0.50	-		2.40	0.80	-	
Vitamin D, μg/d	3.7	1.3	-		4.2	1.5	-		5.1	1.3	-		5.4	1.8	-		6.3	2.3	-	^e^	6.6	2.0	-	^e^	8.0	2.9	-	^e^
Vitamin E, mg/d	4.5	1.1	-		5.4	1.1	-		6.1	1.1	-		6.9	1.1	-		7.6	1.7	-		9.3	2.0	-		10.0	2.6	-	
Vitamin K, μg/d	129	56	-		155	63	-		154	51	-		181	29	-		196	65	-		232	92	-		262	101	-	
Pantothenic acid, mg/d	3.90	0.90	-		4.70	0.90	-		5.20	0.80	-		6.10	0.90	-	^e^	6.60	1.50	-		7.90	1.80	-		8.30	2.20	-	
Potassium, mg/d	1563	327	-		1839	361	-		2005	324	-		2337	331	-		2541	604	-		2983	739	-		3012	839	-	
Phosphorus, mg/d	667	145	-		820	157	-		931	129	-		1090	171	-		1185	263	-		1381	296	-		1449	381	-	
Manganese, mg/d	1.60	0.40	-		2.00	0.40	-		2.30	0.40	-		2.70	0.40	-		2.90	0.60	-	^e^	3.80	1.00	-	^e^	4.60	1.40	-	^e^

AI, Adequate Intake; DG, Tentative Dietary Goal for Preventing Lifestyle-related Diseases; DRIs, Dietary Reference Intakes; EAR, Estimated Average Requirement; NE, niacin equivalent; SD, standard deviation; SFA, saturated fatty acid; PUFA, polyunsaturated fatty acid; RAE, retinol active equivalent. ^a^ Usual intake was estimated using the Multiple Source Method [39] based on 8-day weighed dietary-record data. ^b^ Adequacy of nutrient intake was assessed using the DRIs for Japanese, 2020 [30]. Hyphens indicate the absence of a reference value for nutrients with EAR or DG or that the prevalence of inadequacy was not evaluated for others. ^c^ The percentage of participants below the EAR (cut-point method). ^d^ The percentage of participants whose iron intake was considered inadequate (probability approach). ^e^ The median usual intake was below AI.

**Table 3:** Mean (SD) and inadequacy of nutrient intakes from foods and beverages among Japanese female children and adolescents aged 1–17 years (*N* = 807) ^a^.

Variables	1–2 Years				3–5 Years				6–7 Years				8–9 Years				10–11 Years				12–14 Years				15–17 Years			
	(*N* = 142)				(*N* = 217)				(*N* = 108)				(*N* = 72)				(*N* = 67)				(*N* = 95)				(*N* = 106)			
	Mean	SD	Prevalence of Inadequacy (%) ^b^		Mean	SD	Prevalence of Inadequacy (%) ^b^		Mean	SD	Prevalence of Inadequacy (%) ^b^		Mean	SD	Prevalence of Inadequacy (%) ^b^		Mean	SD	Prevalence of Inadequacy (%) ^b^		Mean	SD	Prevalence of Inadequacy (%) ^b^		Mean	SD	Prevalence of Inadequacy (%) ^b^	
**Energy, kcal/d**	1096	163	-		1360	196	-		1592	212	-		1795	258	-		1930	240	-		2083	292	-		1960	326	-	
**Nutrients without DRI values**																												
Fat, g/d	34.2	7.2	-		46.0	9.2	-		55.8	9.8	-		62.5	11.2	-		68.5	11.8	-		75.5	13.5	-		72.1	14.6	-	
SFA, g/d	11.1	2.8	-		14.6	3.2	-		18.3	3.7	-		19.9	4.3	-		22.0	4.5	-		24.2	5.0	-		21.9	4.6	-	
Carbohydrate, g/d	157.2	23.0	-		187.7	25.8	-		214.2	27.9	-		243.3	35.5	-		257.9	29.7	-		274.2	41.1	-		255.8	45.2	-	
**Nutrients with EAR**			**<EAR**				**<EAR**				**<EAR**				**<EAR**				**<EAR**				**<EAR**				**<EAR**	
Protein, g/d	39.2	7.0	0		48.1	8.2	0		57.3	9.4	0		64.0	10.2	0		69.5	9.0	0		75.7	11.0	0		70.9	13.1	0.9	
Vitamin A, μg RAE/d	367	116	9.9		405	119	31.3		461	106	4.6		494	115	9.7		540	110	7.5		546	129	35.8		493	154	56.6	
Thiamine, mg/d	0.60	0.10	7.7		0.70	0.10	24.0		0.80	0.10	19.4		1.00	0.20	13.9		1.00	0.10	14.9		1.10	0.20	51.6		1.00	0.20	50.0	
Riboflavin, mg/d	0.80	0.20	6.3		0.90	0.20	6.5		1.00	0.20	3.7		1.20	0.20	6.9		1.30	0.20	7.5		1.30	0.20	36.8		1.20	0.20	62.3	
Niacin, mg NE/d	15.7	3.1	0		19.7	3.7	0		23.3	4.2	0		26.3	4.9	0		28.8	4.0	0		31.5	5.2	0		30.1	6.2	0	
Vitamin B-6, mg/d	0.70	0.10	1.4		0.80	0.20	1.8		0.90	0.20	1.9		1.10	0.20	9.7		1.20	0.20	16.4		1.30	0.20	10.5		1.20	0.30	23.6	
Vitamin B-12, μg/d	2.8	0.9	0		3.5	1.0	0		4.4	1.3	0		4.7	1.6	0		4.9	1.2	0		5.4	1.4	0		4.8	1.9	2.8	
Folate, μg/d	170	43	2.1		195	46	0.9		224	49	0		250	62	0		273	41	0		292	68	8.4		289	90	10.4	
Vitamin C, mg/d	62	20	7.7		70	20	6.0		74	22	13.0		83	31	20.8		90	20	16.4		94	34	42.1		93	39	52.8	
Sodium, mg	1968	484	-		2479	540	-		2958	493	-		3454	549	-		3573	506	-		3838	629	-		3755	736	-	
Calcium, mg/d	390	123	43.7		427	122	59.4		517	129	33.3		554	108	69.4		611	157	56.7		565	136	83.2		468	130	70.8	
Magnesium, mg/d	141	27	0		163	30	0		190	34	0		214	40	1.4		230	36	7.5		238	40	53.7		222	52	79.2	
Iron, mg/d	3.9	0.7	11.3	^c^	4.7	1.0	23.5	^c^	5.5	1.0	13.0	^c^	6.2	1.2	43.1	^c^	6.6	1.0	70.1	^c^	7.3	1.2	45.3	^c^	6.9	1.5	18.9	^c^
	-	-	-		-	-	-		-	-	-		-	-	-		-	-	98.5	^d^	-	-	98.9	^d^	-	-	86.8	^d^
	-	-	34.5	^e^	-	-	61.3	^e^	-	-	23.1	^e^	-	-	16.7	^e^	-	-	-	^e^	-	-	92.6	^e^	-	-	91.5	^e^
Zinc, mg/d	4.7	0.8	0		5.8	1.0	0		7.0	1.1	0		7.7	1.3	0		8.4	0.9	0		9.2	1.3	5.3		8.6	1.6	16.0	
Copper, mg/d	0.60	0.10	0		0.70	0.10	0		0.80	0.10	0		0.90	0.20	0		1.00	0.10	0		1.10	0.20	0		1.00	0.20	0	
**Nutrients with DG**			**<DG**	**>DG**			**<DG**	**>DG**			**<DG**	**>DG**			**<DG**	**>DG**			**<DG**	**>DG**			**<DG**	**>DG**			**<DG**	**>DG**
Protein, % energy	14.3	1.4	16.2	0	14.2	1.1	16.1	0	14.4	1.0	7.4	0	14.3	1.2	12.5	0	14.5	1.2	7.5	0	14.6	1.1	7.4	0	14.6	1.5	15.1	0
Fat, % energy	27.6	3.3	1.4	21.1	29.9	3.0	0	49.3	31.2	2.6	0	66.7	31.0	2.5	0	62.5	31.5	2.1	0	80.6	32.2	2.9	0	74.7	32.5	3.2	0	82.1
SFA, % energy	8.9	1.5	-	-	9.5	1.4	-	35.5	10.2	1.2	-	55.6	9.8	1.3	-	48.6	10.1	1.3	-	47.8	10.3	1.2	-	55.8	9.8	1.2	-	92.5
Carbohydrate, % energy	57.9	3.8	0.7	3.5	55.7	3.6	4.6	0.5	54.1	3.4	12.0	0	54.5	3.0	5.6	0	53.8	2.8	4.5	0	52.9	3.4	22.1	0	52.7	3.9	26.4	0
Dietary fibre, g/d	10.5	2.2	-	-	12.6	2.5	1.8	-	14.8	2.4	1.9	-	16.9	3.1	1.4	-	18.4	2.4	1.5	-	19.0	3.1	21.1	-	17.9	4.0	53.8	-
Sodium, g salt equivalent/d	5.0	1.2	-	96.5	6.3	1.4	-	99.1	7.5	1.3	-	100	8.8	1.4	-	100	9.1	1.3	-	100	9.7	1.6	-	96.8	9.5	1.9	-	97.2
Potassium, mg/d	1483	308	-	0	1706	335	19.8	0	1939	355	36.1	0	2173	392	36.1	0	2355	361	14.9	0	2421	419	47.4	0	2214	575	76.4	0
**Nutrients with AI**																												
*n*-6 PUFA, g/d	5.00	1.00	-		6.60	1.30	-		8.00	1.50	-		9.10	1.60	-		10.00	2.00	-		10.80	1.80	-		10.70	2.30	-	
*n*-3 PUFA, g/d	0.90	0.30	-		1.20	0.30	-		1.30	0.30	-		1.60	0.30	-		1.60	0.40	-	^f^	1.80	0.40	-		1.80	0.60	-	
Vitamin D, μg/d	3.6	1.1	-		4.4	1.6	-		4.7	1.2	-	^f^	5.0	1.9	-	^f^	6.0	2.3	-	^f^	6.3	2.0	-	^f^	6.3	2.6	-	^f^
Vitamin E, mg/d	4.1	0.9	-		5.2	1.0	-		5.8	1.1	-		6.5	1.1	-		7.0	1.3	-		7.8	1.5	-		7.6	1.6	-	
Vitamin K, μg/d	133	54	-		139	53	-		156	47	-		172	60	-		185	40	-		203	57	-		209	79	-	
Pantothenic acid, mg/d	3.70	0.70	-	^f^	4.30	0.80	-		5.00	0.90	-	^f^	5.50	0.90	-		6.00	0.80	-	^f^	6.20	1.00	-		5.70	1.10	-	^f^
Potassium, mg/d	1483	308	-		1706	335	-		1939	355	-		2173	392	-		2355	361	-		2421	419	-		2214	575	-	
Phosphorus, mg/d	629	126	-		744	135	-		883	153	-		974	153	-	^f^	1061	160	-		1097	175	-		999	199	-	
Manganese, mg/d	1.50	0.30	-	^f^	1.80	0.40	-		2.20	0.40	-		2.60	0.70	-	^f^	2.80	0.50	-	^f^	3.10	0.70	-	^f^	3.10	0.80	-	^f^

AI, Adequate Intake; DG, Tentative Dietary Goal for Preventing Lifestyle-related Diseases; DRIs, Dietary Reference Intakes; EAR, Estimated Average Requirement; NE, niacin equivalent; SD, standard deviation; SFA, saturated fatty acid; PUFA, polyunsaturated fatty acid; RAE, retinol active equivalent. ^a^ Usual intake was estimated using the Multiple Source Method [39] based on 8-day weighed dietary-record data. ^b^ Adequacy of nutrient intake was assessed using the DRIs for Japanese, 2020 [30]. Hyphens indicate the absence of a reference value for nutrients with EAR or DG or that the prevalence of inadequacy was not evaluated for others. ^c^ The percentage of participants below the EAR for non-menstruating females (cut-point method). ^d^ The percentage of participants below the EAR for menstruating females (cut-point method). ^e^ The percentage of participants whose iron intake was considered inadequate (probability approach). ^f^ The median usual intake was below AI.

**Table 4:** Mean (SD) and inadequacy of nutrient intakes from foods and beverages among Japanese male adults aged 18–79 years (*N* = 1375) ^a^.

Variables	18–29 Years				30–49 Years				50–64 Years				65–74 Years				75–79 Years			
	(*N* = 271)				(*N* = 439)				(*N* = 336)				(*N* = 229)				(*N* = 100)			
	Mean	SD	Prevalence of Inadequacy (%) ^b^		Mean	SD	Prevalence of Inadequacy (%) ^b^		Mean	SD	Prevalence of Inadequacy (%) ^b^		Mean	SD	Prevalence of Inadequacy (%) ^b^		Mean	SD	Prevalence of Inadequacy (%) ^b^	
**Energy, kcal/d**	2205	516	-		2150	465	-		2276	428	-		2223	375	-		2254	406	-	
**Nutrients without DRI values**																				
Fat, g/d	75.2	20.9	-		70.7	19.4	-		72.1	17.3	-		68.8	16.1	-		67.6	19.4	-	
SFA, g/d	22.0	6.9	-		20.2	6.2	-		20.0	5.6	-		18.9	5.0	-		18.3	5.6	-	
Carbohydrate, g/d	296.6	73.4	-		280.3	63.8	-		286.8	57.6	-		285.0	52.7	-		295.1	54.4	-	
**Nutrients with EAR**			**<EAR**				**<EAR**				**<EAR**				**<EAR**				**<EAR**	
Protein, g/d	78.0	21.0	6.3		75.8	17.8	6.2		83.7	17.3	0.9		84.6	14.9	1.3		86.3	19.0	2.0	
Vitamin A, μg RAE/d	446	173	80.8		480	191	83.1		567	214	71.7		659	234	41.9		640	217	37.0	
Thiamine, mg/d	1.10	0.30	60.1		1.10	0.30	68.6		1.20	0.30	44.0		1.20	0.30	41.9		1.20	0.30	33.0	
Riboflavin, mg/d	1.20	0.30	60.5		1.30	0.30	59.2		1.40	0.30	29.5		1.50	0.30	17.0		1.50	0.40	7.0	
Niacin, mg NE/d	34.4	10.0	0.7		34.5	8.7	0.2		38.8	8.9	0		38.5	8.0	0		39.1	10.4	0	
Vitamin B-6, mg/d	1.30	0.40	31.7		1.30	0.40	30.1		1.50	0.40	15.5		1.60	0.40	6.1		1.70	0.50	7.0	
Vitamin B-12, μg/d	5.0	2.2	3.0		5.5	2.2	2.1		7.6	2.9	0		8.7	3.3	0		9.3	3.8	0	
Folate, μg/d	292	105	16.6		310	101	12.3		371	110	2.7		439	121	0.4		432	137	2.0	
Vitamin C, mg/d	87	40	56.5		88	37	54.0		112	44	33.0		140	44	8.7		144	59	9.0	
Sodium, mg	4150	1001	0		4104	869	0		4482	1000	0		4649	1046	0		4617	984	0	
Calcium, mg/d	447	171	87.5		459	157	82.2		540	170	69.3		608	171	50.2		635	209	51.0	
Magnesium, mg/d	241	67	76.4		257	68	81.3		305	74	55.4		326	73	31.9		334	86	20.0	
Iron, mg/d ^c^	7.5	2.0	33.6		7.6	2.1	29.4		8.8	2.2	13.4		9.6	2.1	2.2		9.7	2.5	3.0	
Zinc, mg/d	9.6	2.6	43.9		9.1	2.3	49.9		9.6	2.1	38.4		9.5	1.8	42.8		9.7	2.1	38.0	
Copper, mg/d	1.10	0.30	4.4		1.10	0.30	4.3		1.30	0.30	1.5		1.40	0.30	0.4		1.40	0.30	2.0	
**Nutrients with DG**			**<DG**	**>DG**			**<DG**	**>DG**			**<DG**	**>DG**			**<DG**	**>DG**			**<DG**	**>DG**
Protein, % energy	14.3	1.9	26.2	1.1	14.2	1.9	23.0	0.7	14.8	1.7	31.5	0.6	15.3	1.4	40.6	0.4	15.4	1.6	43.0	0
Fat, % energy	30.0	4.2	0.7	51.3	29.1	4.7	2.5	42.8	28.1	4.1	2.7	34.2	27.5	4.1	3.1	23.1	26.4	4.7	5.0	24.0
SFA, % energy	8.8	1.6	-	90.0	8.3	1.7	-	78.6	7.8	1.6	-	68.5	7.6	1.5	-	62.9	7.2	1.6	-	48.0
Carbohydrate, % energy	54.5	5.5	16.6	1.8	52.9	6.2	29.8	2.3	51.2	6.7	38.4	1.5	51.9	6.5	39.3	1.3	53.1	6.4	31.0	1.0
Dietary fibre, g/d	19.5	5.2	64.9	-	19.6	4.7	63.8	-	21.9	5.4	44.9	-	24.1	5.2	21.4	-	24.2	5.9	22.0	-
Sodium, g salt equivalent/d	10.5	2.5	-	90.8	10.4	2.2	-	90.9	11.4	2.5	-	94.6	11.8	2.7	-	96.1	11.7	2.5	-	99.0
Potassium, mg/d	2323	695	84.1	0	2396	643	85.0	0	2836	717	60.7	0	3113	750	48.9	0	3188	925	51.0	0
**Nutrients with AI**																				
*n*-6 PUFA, g/d	11.30	3.10	-		11.10	3.20	-		11.70	3.00	-		11.10	2.90	-		11.20	3.60	-	
*n*-3 PUFA, g/d	1.90	0.70	-	^d^	2.10	0.70	-		2.50	0.80	-		2.80	0.90	-		2.90	0.90	-	
Vitamin D, μg/d	5.4	2.7	-	^d^	6.1	3.0	-	^d^	8.5	3.6	-	^d^	9.8	3.2	-		11.9	4.5	-	
Vitamin E, mg/d	7.8	2.2	-		7.8	2.2	-		8.6	2.2	-		9.1	2.3	-		9.0	2.6	-	
Vitamin K, μg/d	214	96	-		219	93	-		261	109	-		318	126	-		306	145	-	
Pantothenic acid, mg/d	6.00	1.70	-		5.90	1.50	-		6.50	1.50	-		6.80	1.40	-		7.00	1.90	-	
Potassium, mg/d	2323	695	-	^d^	2396	643	-	^d^	2836	717	-		3113	750	-		3188	925	-	
Phosphorus, mg/d	1052	291	-		1045	257	-		1183	260	-		1225	238	-		1252	302	-	
Manganese, mg/d	3.80	1.50	-	^d^	3.70	1.20	-	^d^	4.20	1.20	-		4.60	1.60	-		4.80	1.70	-	

AI, Adequate Intake; DG, Tentative Dietary Goal for Preventing Lifestyle-related Diseases; DRIs, Dietary Reference Intakes; EAR, Estimated Average Requirement; NE, niacin equivalent; SD, standard deviation; SFA, saturated fatty acid; PUFA, polyunsaturated fatty acid; RAE, retinol active equivalent. ^a^ Usual intake was estimated using the Multiple Source Method [39] based on 8-day weighed dietary-record data. ^b^ Adequacy of nutrient intake was assessed using the DRIs for Japanese, 2020 [30]. Hyphens indicate the absence of a reference value for nutrients with EAR or DG or that the prevalence of inadequacy was not evaluated for others. ^c^ The percentage of participants below the EAR (cut-point method). ^d^ The median usual intake was below AI.

**Table 5:** Mean (SD) and inadequacy of nutrient intakes from foods and beverages among Japanese female adults aged 18–79 years (*N* = 1427) ^a^.

Variables	18–29 Years				30–49 Years				50–64 Years				65–74 Years				75–79 Years			
	(*N* = 291)				(*N* = 443)				(*N* = 362)				(*N* = 243)				(*N* = 88)			
	Mean	SD	Prevalence of Inadequacy (%) ^b^		Mean	SD	Prevalence of Inadequacy (%) ^b^		Mean	SD	Prevalence of Inadequacy (%) ^b^		Mean	SD	Prevalence of Inadequacy (%) ^b^		Mean	SD	Prevalence of Inadequacy (%) ^b^	
**Energy, kcal/d**	1671	290	-		1782	320	-		1871	287	-		1900	297	-		1797	324	-	
**Nutrients without DRI values**																				
Fat, g/d	58.7	13.6	-		60.8	13.5	-		64.2	12.5	-		63.4	13.4	-		55.7	13.4	-	
SFA, g/d	17.5	4.6	-		18.1	4.7	-		18.6	4.1	-		17.8	4.4	-		15.6	4.3	-	
Carbohydrate, g/d	220.9	41.3	-		233.0	45.1	-		242.6	43.4	-		250.1	44.2	-		249.6	45.0	-	
**Nutrients with EAR**			**<EAR**				**<EAR**				**<EAR**				**<EAR**				**<EAR**	
Protein, g/d	61.3	11.7	3.1		64.9	12.2	2.0		71.1	11.9	0.3		76.4	14.0	0		71.4	14.3	1.1	
Vitamin A, μg RAE/d	434	155	58.1		494	172	55.3		578	173	34.5		693	273	20.6		652	213	18.2	
Thiamine, mg/d	0.90	0.20	54.6		0.90	0.20	47.9		1.00	0.20	26.5		1.10	0.20	21.4		1.00	0.30	28.4	
Riboflavin, mg/d	1.00	0.20	46.7		1.10	0.30	30.9		1.30	0.30	10.5		1.50	0.30	7.8		1.40	0.40	10.2	
Niacin, mg NE/d	26.6	5.8	0		29.1	6.1	0		32.4	6.1	0		34.4	6.9	0		31.2	6.4	0	
Vitamin B-6, mg/d	1.00	0.30	46.7		1.20	0.30	28.2		1.30	0.30	13.8		1.50	0.40	7.0		1.40	0.30	12.5	
Vitamin B-12, μg/d	4.1	1.6	4.1		4.7	2.1	3.6		6.0	2.0	0.3		7.7	3.3	0		7.2	2.6	0	
Folate, μg/d	268	85	22.0		308	96	8.8		372	100	2.5		435	132	0.4		401	124	1.1	
Vitamin C, mg/d	81	32	59.5		96	35	40.6		125	41	17.7		152	53	7.8		141	51	8.0	
Sodium, mg	3354	678	0		3520	694	0		3886	702	0		4140	909	0		4035	942	0	
Calcium, mg/d	404	131	86.3		463	139	78.3		540	140	56.4		621	181	36.6		582	174	33.0	
Magnesium, mg/d	205	53	74.6		236	56	57.8		276	59	26.8		308	75	14.0		283	62	13.6	
Iron, mg/d	6.4	1.5	29.6	^c^	7.0	1.7	14.2	^c^	8.1	1.6	5.5	^c^	9.2	2.2	0.8	^c^	8.5	2.0	3.4	^c^
	-	-	90.0	^d^	-	-	90.7	^d^	-	-	73.5	^d^	-	-	-		-	-	-	
	-	-	95.2	^e^	-	-	92.3	^e^	-	-	79.3	^e^	-	-	-		-	-	-	
Zinc, mg/d	7.2	1.4	41.9		7.6	1.4	35.2		8.1	1.4	21.8		8.7	1.9	13.2		8.1	1.6	8.0	
Copper, mg/d	0.90	0.20	2.7		1.00	0.20	2.0		1.10	0.20	0.3		1.30	0.30	0		1.20	0.20	0	
**Nutrients with DG**			**<DG**	**>DG**			**<DG**	**>DG**			**<DG**	**>DG**			**<DG**	**>DG**			**<DG**	**>DG**
Protein, % energy	14.8	1.9	14.8	1.4	14.7	1.6	11.3	0.2	15.3	1.6	18.2	1.1	16.2	1.6	23.0	1.2	16.0	1.5	27.3	1.1
Fat, % energy	30.9	3.9	0.7	59.8	30.2	3.5	0.5	54.2	30.4	3.4	0.3	51.7	29.6	3.9	0.4	42.4	27.3	3.4	1.1	20.5
SFA, % energy	9.2	1.5	-	92.8	9.0	1.5	-	89.8	8.8	1.4	-	93.4	8.3	1.5	-	81.5	7.7	1.3	-	71.6
Carbohydrate, % energy	53.6	5.2	22.0	2.4	52.9	5.1	23.3	0.5	52.3	5.2	30.9	0	53.1	4.8	24.3	0	56.0	4.5	8.0	2.3
Dietary fibre, g/d	16.3	3.4	72.2	-	17.8	3.9	53.3	-	20.2	4.1	30.9	-	22.7	5.5	12.3	-	21.7	4.6	18.2	-
Sodium, g salt equivalent/d	8.5	1.7	-	88.7	8.9	1.8	-	92.1	9.9	1.8	-	98.9	10.5	2.3	-	98.4	10.2	2.4	-	95.5
Potassium, mg/d	1996	529	87.6	0	2303	548	72.9	0	2702	617	45.9	0	3100	774	23.9	0	2849	681	37.5	0
**Nutrients with AI**																				
*n*-6 PUFA, g/d	9.00	2.20	-		9.70	2.20	-		10.40	2.10	-		10.40	2.40	-		9.00	1.90	-	
*n*-3 PUFA, g/d	1.50	0.60	-	^f^	1.80	0.60	-		2.10	0.60	-		2.40	0.70	-		2.40	0.90	-	
Vitamin D, μg/d	5.4	2.5	-	^f^	5.6	2.5	-	^f^	7.4	2.6	-	^f^	9.1	3.5	-		9.6	3.8	-	
Vitamin E, mg/d	6.6	1.6	-		7.3	1.8	-		8.3	1.8	-		9.0	2.2	-		8.1	2.0	-	
Vitamin K, μg/d	192	75	-		217	82	-		252	91	-		308	132	-		296	114	-	
Pantothenic acid, mg/d	4.90	1.10	-	^f^	5.30	1.10	-		5.80	1.10	-		6.50	1.40	-		6.10	1.40	-	
Potassium, mg/d	1996	529	-	^f^	2303	548	-		2702	617	-		3100	774	-		2849	681	-	
Phosphorus, mg/d	856	182	-		936	196	-		1045	192	-		1144	238	-		1060	228	-	
Manganese, mg/d	3.00	1.00	-	^f^	3.40	1.60	-	^f^	3.90	1.40	-		4.30	1.30	-		4.00	1.10	-	

AI, Adequate Intake; DG, Tentative Dietary Goal for Preventing Lifestyle-related Diseases; DRIs, Dietary Reference Intakes; EAR, Estimated Average Requirement; NE, niacin equivalent; SD, standard deviation; SFA, saturated fatty acid; PUFA, polyunsaturated fatty acid; RAE, retinol active equivalent. ^a^ Usual intake was estimated using the Multiple Source Method [39] based on 8-day weighed dietary-record data. ^b^ Adequacy of nutrient intake was assessed using the DRIs for Japanese, 2020 [30]. Hyphens indicate the absence of a reference value for EAR or DG or that the prevalence of inadequacy was not evaluated for others. ^c^ The percentage of participants below the EAR for non-menstruating females (cut-point method). ^d^ The percentage of participants below the EAR for menstruating females (cut-point method). ^e^ The percentage of participants whose iron intake was considered inadequate (probability approach). ^f^ The median usual intake was below AI.

## Data Availability

The datasets generated and analyzed during the present study are not publicly available due to restrictions imposed by the Ministry of Health, Labour and Welfare.

## Supplementary Materials

The following supporting information can be downloaded at: https://www.mdpi.com/article/10.3390/nu15245113/s1, Table S1: Dietary Reference Intakes (2020) for Japanese male children and adolescents; Table S2: Dietary Reference Intakes (2020) for Japanese female children and adolescents; Table S3: Dietary Reference Intakes (2020) for Japanese male adults; Table S4: Dietary Reference Intakes (2020) for Japanese female adults; Table S5: Usual intake distribution of energy and nutrients among Japanese aged 1–2 years; Table S6: Usual intake distribution of energy and nutrients among Japanese aged 3–5 years; Table S7: Usual intake distribution of energy and nutrients among Japanese aged 6–7 years; Table S8: Usual intake distribution of energy and nutrients among Japanese aged 8–9 years; Table S9: Usual intake distribution of energy and nutrients among Japanese aged 10–11 years; Table S10: Usual intake distribution of energy and nutrients among Japanese aged 12–14 years; Table S11: Usual intake distribution of energy and nutrients among Japanese aged 15–17 years; Table S12: Usual intake distribution of energy and nutrients among Japanese aged 18–29 years; Table S13: Usual intake distribution of energy and nutrients among Japanese aged 30–49 years; Table S14: Usual intake distribution of energy and nutrients among Japanese aged 50–64 years; Table S15: Usual intake distribution of energy and nutrients among Japanese aged 65–74 years; Table S16: Usual intake distribution of energy and nutrients among Japanese aged 75–79 years.

## References

[1] GBD 2017 Diet Collaborators (2019). Health effects of dietary risks in 195 countries, 1990–2017: A systematic analysis for the Global Burden of Disease Study 2017. Lancet.

[2] Sasaki S. (2020). for Working Group 1 of the Healthy Diet Research Committee of International Life Sciences Institute, Japan. What is the scientific definition of the Japanese diet from the viewpoint of nutrition and health?. Nutr. Rev..

[3] Murakami K., Livingstone M.B.E., Fujiwara A., Sasaki S. (2020). Application of the Healthy Eating Index-2015 and the Nutrient-Rich Food Index 9.3 for assessing overall diet quality in the Japanese context: Different nutritional concerns from the US. PLoS ONE.

[4] Asakura K., Uechi K., Sasaki Y., Masayasu S., Sasaki S. (2014). Estimation of sodium and potassium intakes assessed by two 24 h urine collections in healthy Japanese adults: A nationwide study. Br. J. Nutr..

[5] Ministry of Health, Labour and Welfare National Health and Nutrition Survey 2019. https://www.mhlw.go.jp/stf/seisakunitsuite/bunya/kenkou_iryou/kenkou/eiyou/r1-houkoku_00002.html.

[6] Huang K., Fang H., Yu D., Guo Q., Xu X., Ju L., Cai S., Yang Y., Wei X., Zhao L. (2022). Usual Intake of Micronutrients and Prevalence of Inadequate Intake among Chinese Adults: Data from CNHS 2015–2017. Nutrients.

[7] Kim D.W., Shim J.E., Paik H.Y., Song W.O., Joung H. (2011). Nutritional intake of Korean population before and after adjusting for within-individual variations: 2001 Korean National Health and Nutrition Survey Data. Nutr. Res. Pract..

[8] Huang K., Yu D., Guo Q., Yang Y., Wei X., Zhao L., Fang H. (2022). Validation of the MSM and NCI Method for Estimating the Usual Intake of Nutrients and Food According to Four Seasons of Seven Consecutive Daily 24 Hour Dietary Recalls in Chinese Adults. Nutrients.

[9] Laureano G.H., Torman V.B., Crispim S.P., Dekkers A.L., Camey S.A. (2016). Comparison of the ISU, NCI, MSM, and SPADE Methods for Estimating Usual Intake: A Simulation Study of Nutrients Consumed Daily. Nutrients.

[10] Qin Y., Cowan A.E., Bailey R.L., Jun S., Eicher-Miller H.A. (2023). Usual nutrient intakes and diet quality among United States older adults participating in the Supplemental Nutrition Assistance Program compared with income-eligible nonparticipants. Am. J. Clin. Nutr..

[11] Bailey A.D.L., Fulgoni Iii V.L., Shah N., Patterson A.C., Gutierrez-Orozco F., Mathews R.S., Walsh K.R. (2021). Nutrient Intake Adequacy from Food and Beverage Intake of US Children Aged 1–6 Years from NHANES 2001–2016. Nutrients.

[12] Ahluwalia N., Herrick K.A., Rossen L.M., Rhodes D., Kit B., Moshfegh A., Dodd K.W. (2016). Usual nutrient intakes of US infants and toddlers generally meet or exceed Dietary Reference Intakes: Findings from NHANES 2009-2012. Am. J. Clin. Nutr..

[13] Pedroza-Tobias A., Hernandez-Barrera L., Lopez-Olmedo N., Garcia-Guerra A., Rodriguez-Ramirez S., Ramirez-Silva I., Villalpando S., Carriquiry A., Rivera J.A. (2016). Usual Vitamin Intakes by Mexican Populations. J. Nutr..

[14] Brassard D., Chevalier S. (2023). Relationship between Adherence to the 2019 Canada’s Food Guide Recommendations on Healthy Food Choices and Nutrient Intakes in Older Adults. J. Nutr..

[15] Kehoe L., Buffini M., McNulty B.A., Kearney J.M., Flynn A., Walton J. (2023). Food and nutrient intakes and compliance with recommendations in school-aged children in Ireland: Findings from the National Children’s Food Survey II (2017–2018) and changes since 2003–2004. Br. J. Nutr..

[16] Walton J., Kehoe L., McNulty B.A., Nugent A.P., Flynn A. (2017). Nutrient intakes and compliance with nutrient recommendations in children aged 1–4 years in Ireland. J. Hum. Nutr. Diet..

[17] Bird J.K., Bruins M.J., Turini M.E. (2023). Micronutrient intakes in the Dutch diet: Foods, fortified foods and supplements in a cross sectional study. Eur. J. Nutr..

[18] Valsta L.M., Tapanainen H., Kortetmaki T., Sares-Jaske L., Paalanen L., Kaartinen N.E., Haario P., Kaljonen M. (2022). Disparities in Nutritional Adequacy of Diets between Different Socioeconomic Groups of Finnish Adults. Nutrients.

[19] Gregoric M., Hristov H., Blaznik U., Korousic Seljak B., Delfar N., Pravst I. (2022). Dietary Intakes of Slovenian Adults and Elderly: Design and Results of the National Dietary Study SI.Menu 2017/18. Nutrients.

[20] Mitsopoulou A.V., Magriplis E., Michas G., Micha R., Chourdakis M., Chrousos G.P., Roma E., Panagiotakos D.B., Zampelas A., Karageorgou D. (2021). Micronutrient dietary intakes and their food sources in adults: The Hellenic National Nutrition and Health Survey (HNNHS). J. Hum. Nutr. Diet..

[21] Lopez-Sobaler A.M., Aparicio A., Rubio J., Marcos V., Sanchidrian R., Santos S., Perez-Farinos N., Dal-Re M.A., Villar-Villalba C., Yusta-Boyo M.J. (2019). Adequacy of usual macronutrient intake and macronutrient distribution in children and adolescents in Spain: A National Dietary Survey on the Child and Adolescent Population, ENALIA 2013–2014. Eur. J. Nutr..

[22] Flynn A., Hirvonen T., Mensink G.B., Ocke M.C., Serra-Majem L., Stos K., Szponar L., Tetens I., Turrini A., Fletcher R. (2009). Intake of selected nutrients from foods, from fortification and from supplements in various European countries. Food Nutr. Res..

[23] Liu Z., Zhao L., Man Q., Wang J., Zhao W., Zhang J. (2019). Dietary Micronutrients Intake Status among Chinese Elderly People Living at Home: Data from CNNHS 2010–2012. Nutrients.

[24] Angeles-Agdeppa I., Sun Y., Denney L., Tanda K.V., Octavio R.A.D., Carriquiry A., Capanzana M.V. (2019). Food sources, energy and nutrient intakes of adults: 2013 Philippines National Nutrition Survey. Nutr. J..

[25] Ikeda N., Takimoto H., Imai S., Miyachi M., Nishi N. (2015). Data Resource Profile: The Japan National Health and Nutrition Survey (NHNS). Int. J. Epidemiol..

[26] Murakami K., Okubo H., Livingstone M.B.E., Fujiwara A., Asakura K., Uechi K., Sugimoto M., Wang H.C., Masayasu S., Sasaki S. (2018). Adequacy of Usual Intake of Japanese Children Aged 3–5 Years: A Nationwide Study. Nutrients.

[27] Sugimoto M., Murakami K., Fujiwara A., Asakura K., Masayasu S., Sasaki S. (2020). Association between diet-related greenhouse gas emissions and nutrient intake adequacy among Japanese adults. PLoS ONE.

[28] Asakura K., Sasaki S. (2017). School lunches in Japan: Their contribution to healthier nutrient intake among elementary-school and junior high-school children. Public Health Nutr..

[29] Tokudome Y.I.N., Nagaya T., Ikeda M., Fujiwara N., Sato J., Kuriki K., Kikuchi S., Maki S., Tokudome S. (2002). Daily, weekly, seasonal, within- and between-individual variation in nutrient intake according to four season consecutive 7 day weighed diet records in Japanese female dietitians. J. Epidemiol..

[30] Ministry of Health, Labour and Welfare, Japan (2020). Dietary Reference Intakes for Japanese. https://www.mhlw.go.jp/content/10904750/000586553.pdf.

[31] Murakami K., Livingstone M.B.E., Masayasu S., Sasaki S. (2021). Eating patterns in a nationwide sample of Japanese aged 1–79 years from MINNADE study: Eating frequency, clock time for eating, time spent on eating and variability of eating patterns. Public Health Nutr..

[32] Statistics Bureau & Ministry of Internal Affairs and Communications (2015) Population and Households of Japan 2015. https://www.stat.go.jp/english/data/kokusei/2015/poj/mokuji.html.

[33] Ministry of Education, Culture, Sports, Science and Technology (MEXT), Japan (2020). Standard Tables of Food Composition in Japan.

[34] Huang T.T.K., Roberts S.B., Howarth N.C., McCrory M.A. (2005). Effect of screening out implausible energy intake reports on relationships between diet and BMI. Obes. Res..

[35] National Academies of Sciences, Engineering, and Medicine, Health and Medicine Division, Food and Nutrition Board, Committee on the Dietary Reference Intakes for Energy (2023). Dietary Reference Intakes for Energy.

[36] Huang T.T., Howarth N.C., Lin B.H., Roberts S.B., McCrory M.A. (2004). Energy intake and meal portions: Associations with BMI percentile in U.S. children. Obes. Res..

[37] Livingstone M.B.E., Black A.E. (2003). Markers of the Validity of Reported Energy Intake. J. Nutr..

[38] European Food Safety Authority (2014). Guidance on the EU Menu methodology. EFSA J..

[39] Harttig U., Haubrock J., Knuppel S., Boeing H., Consortium E. (2011). The MSM program: Web-based statistics package for estimating usual dietary intake using the Multiple Source Method. Eur. J. Clin. Nutr..

[40] Murphy S.P., Guenther P.M., Kretsch M.J. (2006). Using the dietary reference intakes to assess intakes of groups: Pitfalls to avoid. J. Am. Diet. Assoc..

[41] Trumbo P.R., Barr S.I., Murphy S.P., Yates A.A. (2013). Dietary reference intakes: Cases of appropriate and inappropriate uses. Nutr. Rev..

[42] WHO/FAO (2006). Guidelines on Food Fortification with Micronutrients.

[43] Murphy S.P., Barr S.I. (2011). Practice paper of the American Dietetic Association: Using the Dietary Reference Intakes. J. Am. Diet. Assoc..

[44] Institute of Medicine (2006). Dietary Reference Intakes: The Essential Guide to Nutrient Requirements.

[45] (2023). Saturated Fatty Acid and Trans-Fatty Acid Intake for Adults and Children: WHO Guideline.

[46] Krauss R.M., Kris-Etherton P.M. (2020). Public health guidelines should recommend reducing saturated fat consumption as much as possible: Debate Consensus. Am. J. Clin. Nutr..

[47] Astrup A., Magkos F., Bier D.M., Brenna J.T., de Oliveira Otto M.C., Hill J.O., King J.C., Mente A., Ordovas J.M., Volek J.S. (2020). Saturated Fats and Health: A Reassessment and Proposal for Food-Based Recommendations: JACC State-of-the-Art Review. J. Am. Coll. Cardiol..

[48] Morgan P.T., Witard O.C., Hojfeldt G., Church D.D., Breen L. (2023). Dietary protein recommendations to support healthy muscle ageing in the 21st century and beyond: Considerations and future directions. Proc. Nutr. Soc..

[49] Miketinas D.C., Tucker W.J., Douglas C.C., Patterson M.A. (2023). Usual dietary fibre intake according to diabetes status in USA adults-NHANES 2013–2018. Br. J. Nutr..

[50] The Council for Science and Technology (2010). Standard Tables of Food Composition in Japan, Fifth Revised and Enlarged Edition.

[51] Council for Science and Technology, Ministry of Education, Culture, Sports, Science and Technology, Japan (2015). Standard Tables of Food Composition in Japan 2015.

[52] Koyama T., Yamaoka S. (2022). Changes in the intake of dietary fibre derived from rice among Japanese people based on the National Health and Nutrition Survey during 2018–2019. Integr. Food Nutr. Metab..

[53] O’Neil C.E., Keast D.R., Fulgoni V.L., Nicklas T.A. (2012). Food sources of energy and nutrients among adults in the US: NHANES 2003–2006. Nutrients.

[54] Cifelli C.J., Agarwal S., Fulgoni V.L. (2022). Association between Intake of Total Dairy and Individual Dairy Foods and Markers of Folate, Vitamin B(6) and Vitamin B(12) Status in the U.S. Population. Nutrients.

[55] Lim K.H., Riddell L.J., Nowson C.A., Booth A.O., Szymlek-Gay E.A. (2013). Iron and zinc nutrition in the economically-developed world: A review. Nutrients.

[56] Petraglia F., Dolmans M.M. (2022). Iron deficiency anemia: Impact on women’s reproductive health. Fertil. Steril..

[57] WHO (2012). Guideline: Sodium Intake for Adults and Children.

[58] Asakura K., Uechi K., Masayasu S., Sasaki S. (2016). Sodium sources in the Japanese diet: Difference between generations and sexes. Public Health Nutr..

[59] Willey J., Gardener H., Cespedes S., Cheung Y.K., Sacco R.L., Elkind M.S.V. (2017). Dietary Sodium to Potassium Ratio and Risk of Stroke in a Multiethnic Urban Population: The Northern Manhattan Study. Stroke.

[60] Okayama A., Okuda N., Miura K., Okamura T., Hayakawa T., Akasaka H., Ohnishi H., Saitoh S., Arai Y., Kiyohara Y. (2016). Dietary sodium-to-potassium ratio as a risk factor for stroke, cardiovascular disease and all-cause mortality in Japan: The NIPPON DATA80 cohort study. BMJ Open.

[61] Matsuda-Inoguchi N., Date C., Sakurai K., Kuwazoe M., Watanabe T., Toji C., Furukawa Y., Shimbo S., Nakatsuka H., Ikeda M. (2006). Reduction in estimated vitamin A intake induced by new food composition tables in Japan, where vitamin A is taken mostly from plant foods. Int. J. Food Sci. Nutr..

[62] Yamada M., Asakura K., Sasaki S., Hirota N., Notsu A., Todoriki H., Miura A., Fukui M., Date C. (2014). Estimation of intakes of copper, zinc, and manganese in Japanese adults using 16-day semi-weighed diet records. Asia Pac. J. Clin. Nutr..

[63] Chen G., Weiskirchen S., Weiskirchen R. (2023). Vitamin A: Too good to be bad?. Front. Pharmacol..

[64] Martins A.C., Krum B.N., Queiros L., Tinkov A.A., Skalny A.V., Bowman A.B., Aschner M. (2020). Manganese in the Diet: Bioaccessibility, Adequate Intake, and Neurotoxicological Effects. J. Agric. Food Chem..

[65] Ministry of Health, Labour and Welfare Comprehensive Survey of Living Conditions 2020. https://www.mhlw.go.jp/toukei/saikin/hw/k-tyosa/k-tyosa19/index.html.

[66] Nishijima C., Sato Y., Chiba T. (2023). Nutrient Intake from Voluntary Fortified Foods and Dietary Supplements in Japanese Consumers: A Cross-Sectional Online Survey. Nutrients.

[67] Kobayashi M., Adachi H.Y., Ishihara J., Tsugane S., Group J.F.V.S. (2011). Effect of cooking loss in the assessment of vitamin intake for epidemiological data in Japan. Eur. J. Clin. Nutr..

