# Supplementary material for: Usual Nutrient Intake Distribution and Prevalence of Nutrient Intake Inadequacy among Japanese Children and Adults: A Nationwide Study Based on 8-Day Dietary Records
Source: Nutrients. 2023 Dec 14;15(24):5113. doi: 10.3390/nu15245113 (PMC10746136; doi:10.3390/nu15245113)
Supplement: Supplementary file 1 [file nutrients-15-05113-s001.zip › nutrients-2768050-supplementary.pdf]

Table S1. Dietary Reference Intakes (2020) for Japanese male children and adolescents [a].

Variables	1–2 years	3–5 years	6–7 years	8–9 years	10–11 years	12–14 years	15–17 years
**Energy, kcal/d** [b]							
Phisical activity level Ⅰ	-	-	1350	1600	1950	2300	2500
Phisical activity level Ⅱ	950	1300	1550	1850	2250	2600	2800
Phisical activity level Ⅲ	-	-	1750	2100	2500	2900	3150
**Nutrients with EAR**							
Protein, g/d	15	20	25	30	40	50	50
Vitamin A, μg RAE/d	300	350	300	350	450	550	650
Thiamine, mg/d	0.4	0.6	0.7	0.8	1	1.2	1.3
Riboflavin, mg/d	0.5	0.7	0.8	0.9	1.1	1.3	1.4
Niacin, mg NE/d	5	6	7	9	11	12	14
Vitamin B-6, mg/d	0.4	0.5	0.7	0.8	1	1.2	1.2
Vitamin B-12, μg/d	0.8	0.9	1.1	1.3	1.6	2	2
Folate, μg/d	80	90	110	130	160	200	220
Vitamin C, mg/d	35	40	50	60	70	85	85
Sodium, mg/d	-	-	-	-	-	-	-
Calcium, mg/d	350	500	500	550	600	850	650
Magnesium, mg/d	60	80	110	140	180	250	300
Iron, mg/d	3	4	5	6	7	8	8
Zinc, mg/d	3	3	4	5	6	9	10
Copper, mg/d	0.3	0.3	0.4	0.4	0.5	0.7	0.8
**Nutrients with DG**							
Protein, % energy	13–20	13–20	13–20	13–20	13–20	13–20	13–20
Fat, % energy	20–30	20–30	20–30	20–30	20–30	20–30	20–30
SFA, % energy	-	≤10	≤10	≤10	≤10	≤10	≤8
Carbohydrate, % energy	50–65	50–65	50–65	50–65	50–65	50–65	50–65
Dietary fibre, g/d	-	≥8	≥10	≥11	≥13	≥17	≥19
Sodium, g salt equivalent/d	<3	<3.5	<4.5	<5	<6	<7	<7.5
Potassium, mg/d	-	≥1400	≥1800	≥2000	≥2200	≥2400	≥3000
**Nutrients with AI**							
n-6 PUFA, g/d	4	6	8	8	10	11	13
n-3 PUFA, g/d	0.7	1.1	1.5	1.5	1.6	1.9	2.1
Vitamin D, μg/d	3	3.5	4.5	5	6.5	8	9
Vitamin E, mg/d	3	4	5	5	5.5	6.5	7
Vitamin K, μg/d	50	60	80	90	110	140	160
Pantothenic acid, mg/d	3	4	5	6	6	7	7
Potassium, mg/d	900	1000	1300	1500	1800	2300	2700
Phosphorus, mg/d	500	700	900	1000	1100	1200	1200
Manganese, mg/d	1.5	1.5	2	2.5	3	4	4.5
**Nutrients with UL**							
Vitamin A, μg RAE/d	600	700	950	1200	1500	2100	2500
Vitamin D, μg/d	20	30	30	40	60	80	90
Vitamin E, mg/d	150	200	300	350	450	650	750
Niacin, mg NE/d [c]	60 (15)	80 (20)	100 (30)	150 (35)	200 (45)	250 (60)	300 (70)
Vitamin B-6, mg/d	10	15	20	25	30	40	50
Folate, μg/d	200	300	400	500	700	900	900
Calcium, mg/d	-	-	-	-	-	-	-
Phosphorus, mg/d	-	-	-	-	-	-	-
Iron, mg/d	25	25	30	35	35	40	50
Zinc, mg/d	-	-	-	-	-	-	-
Copper, mg/d	-	-	-	-	-	-	-
Manganese, mg/d	-	-	-	-	-	-	-

AI, Adequate Intake; DG, Tentative Dietary Goal for Preventing Lifestyle-related Diseases; EAR, Estimated Average Requirement; SD, standard deviation; SFA, saturated fatty acid; PUFA, polyunsaturated fatty acid. [a] Hyphens indicate the absence of a reference value. [b] The reference values for energy were not considered in this study. [c] The amount of nicotinamide. The values in parentheses are the amount of nicotinic acid.

Table S2. Dietary Reference Intakes (2020) for Japanese female children and adolescents [a].

Variables	1–2 years	3–5 years	6–7 years	8–9 years	10–11 years	12–14 years	15–17 years
**Energy, kcal/d** [b]							
Phisical activity level Ⅰ	-	-	1250	1500	1850	2150	2050
Phisical activity level Ⅱ	900	1250	1450	1700	2100	2400	2300
Phisical activity level Ⅲ	-	-	1650	1900	2350	2700	2550
**Nutrients with EAR**							
Protein, g/d	15	20	25	30	40	45	45
Vitamin A, μg RAE/d	250	350	300	350	400	500	500
Thiamine, mg/d	0.4	0.6	0.7	0.8	0.9	1.1	1
Riboflavin, mg/d	0.5	0.6	0.7	0.9	1	1.2	1.2
Niacin, mg NE/d	4	6	7	8	10	12	11
Vitamin B-6, mg/d	0.4	0.5	0.6	0.8	1	1	1
Vitamin B-12, μg/d	0.8	0.9	1.1	1.3	1.6	2	2
Folate, μg/d	90	90	110	130	160	200	200
Vitamin C, mg/d	35	40	50	60	70	85	85
Sodium, mg/d	-	-	-	-	-	-	-
Calcium, mg/d	350	450	450	600	600	700	550
Magnesium, mg/d	60	80	110	140	180	240	260
Iron, mg/d (for non-menstruating females)	3	4	4.5	6	7	7	5.5
Iron, mg/d (for menstruating females)	-	-	-	-	10	10	8.5
Zinc, mg/d	2	3	3	4	5	7	7
**Nutrients with DG**							
Copper, mg/d	≥0.2	≥0.3	≥0.4	≥0.4	≥0.5	≥0.6	≥0.6
Protein, % energy	13–20	13–20	13–20	13–20	13–20	13–20	13–20
Fat, % energy	20–30	20–30	20–30	20–30	20–30	20–30	20–30
SFA, % energy	-	≤10	≤10	≤10	≤10	≤10	≤8
Carbohydrate, % energy	50–65	50–65	50–65	50–65	50–65	50–65	50–65
Dietary fibre, g/d	-	≥8	≥10	≥11	≥13	≥17	≥18
Sodium, g salt equivalent/d	<3	<3.5	<4.5	<5	<6	<6.5	<6.5
**Nutrients with AI**							
Potassium, mg/d	-	1400	1800	2000	2000	2400	2600
n-6 PUFA, g/d	4	6	7	7	8	9	9
n-3 PUFA, g/d	0.8	1	1.3	1.3	1.6	1.6	1.6
Vitamin D, μg/d	3.5	4	5	6	8	9.5	8.5
Vitamin E, mg/d	3	4	5	5	5.5	6	5.5
Vitamin K, μg/d	60	70	90	110	140	170	150
Pantothenic acid, mg/d	4	4	5	5	6	6	6
Potassium, mg/d	900	1000	1200	1500	1800	1900	2000
Phosphorus, mg/d	500	700	800	1000	1000	1000	900
**Nutrients with UL**							
Manganese, mg/d	1.5	1.5	2	2.5	3	4	3.5
Vitamin A, μg RAE/d	600	850	1200	1500	1900	2500	2800
Vitamin D, μg/d	20	30	30	40	60	80	90
Vitamin E, mg/d	150	200	300	350	450	600	650
Niacin, mg NE/d [c]	60 (15)	80 (20)	100 (30)	150 (35)	150 (45)	250 (60)	250 (65)
Vitamin B-6, mg/d	10	15	20	25	30	40	45
Folate, μg/d	200	300	400	500	700	900	900
Calcium, mg/d	-	-	-	-	-	-	-
Phosphorus, mg/d	-	-	-	-	-	-	-
Iron, mg/d	20	25	30	35	35	40	40
Zinc, mg/d	-	-	-	-	-	-	-
Copper, mg/d	-	-	-	-	-	-	-
Manganese, mg/d	-	-	-	-	-	-	-

AI, Adequate Intake; DG, Tentative Dietary Goal for Preventing Lifestyle-related Diseases; EAR, Estimated Average Requirement; SD, standard deviation; SFA, saturated fatty acid; PUFA, polyunsaturated fatty acid. [a] Hyphens indicate the absence of a reference value. [b] The reference values for energy were not considered in this study. [c] The amount of nicotinamide. The values in parentheses are the amount of nicotinic acid.

Table S3. Dietary Reference Intakes (2020) for Japanese male adults [a].

Variables	18–29 years	30–49 years	50–64 years	65–74 years	75–79 years
**Energy, kcal/d** [b]					
Phisical activity level Ⅰ	2300	2300	2200	2050	1800
Phisical activity level Ⅱ	2650	2700	2600	2400	2100
Phisical activity level Ⅲ	3050	3050	2950	2750	-
**Nutrients with EAR**					
Protein, g/d	50	50	50	50	50
Vitamin A, μg RAE/d	600	650	650	600	550
Thiamine, mg/d	1.2	1.2	1.1	1.1	1
Riboflavin, mg/d	1.3	1.3	1.2	1.2	1.1
Niacin, mg NE/d	13	13	12	12	11
Vitamin B-6, mg/d	1.1	1.1	1.1	1.1	1.1
Vitamin B-12, μg/d	2	2	2	2	2
Folate, μg/d	200	200	200	200	200
Vitamin C, mg/d	85	85	85	80	80
Sodium, mg/d	600	600	600	600	600
Calcium, mg/d	650	600	600	600	600
Magnesium, mg/d	280	310	310	290	270
Iron, mg/d	6.5	6.5	6.5	6	6
Zinc, mg/d	9	9	9	9	9
Copper, mg/d	0.7	0.7	0.7	0.7	0.7
**Nutrients with DG**					
Protein, % energy	13–20	13–20	14–20	15–20	15–20
Fat, % energy	20–30	20–30	20–30	20–30	20–30
SFA, % energy	≤7	≤7	≤7	≤7	≤7
Carbohydrate, % energy	50–65	50–65	50–65	50–65	50–65
Dietary fibre, g/d	≥21	≥21	≥21	≥20	≥20
Sodium, g salt equivalent/d	<7.5	<7.5	<7.5	<7.5	<7.5
Potassium, mg/d	≥3000	≥3000	≥3000	≥3000	≥3000
**Nutrients with AI**					
n-6 PUFA, g/d	11	10	10	9	8
n-3 PUFA, g/d	2	2	2.2	2.2	2.1
Vitamin D, μg/d	8.5	8.5	8.5	8.5	8.5
Vitamin E, mg/d	6	6	7	7	6.5
Vitamin K, μg/d	150	150	150	150	150
Pantothenic acid, mg/d	5	5	6	6	6
Potassium, mg/d	2500	2500	2500	2500	2500
Phosphorus, mg/d	1000	1000	1000	1000	1000
Manganese, mg/d	4	4	4	4	4
**Nutrients with UL**					
Vitamin A, μg RAE/d	2700	2700	2700	2700	2700
Vitamin D, μg/d	100	100	100	100	100
Vitamin E, mg/d	850	900	850	850	750
Niacin, mg NE/d [c]	60 (15)	350 (85)	350 (85)	300 (80)	300 (75)
Vitamin B-6, mg/d	55	60	55	50	50
Folate, μg/d	900	1000	1000	900	900
Calcium, mg/d	2500	2500	2500	2500	2500
Phosphorus, mg/d	3000	3000	3000	3000	3000
Iron, mg/d	50	50	50	50	50
Zinc, mg/d	40	45	45	40	40
Copper, mg/d	7	7	7	7	7
Manganese, mg/d	11	11	11	11	11

AI, Adequate Intake; DG, Tentative Dietary Goal for Preventing Lifestyle-related Diseases; EAR, Estimated Average Requirement; SD, standard deviation; SFA, saturated fatty acid; PUFA, polyunsaturated fatty acid. [a] Hyphens indicate the absence of a reference value. [b] The reference values for energy were not considered in this study. [c] The amount of nicotinamide. The values in parentheses are the amount of nicotinic acid.

Table S4. Dietary Reference Intakes (2020) for Japanese female adults [a].

Variables	18–29 years	30–49 years	50–64 years	65–74 years	75–79 years
**Energy, kcal/d** [b]					
Phisical activity level Ⅰ	1700	1750	1650	1550	1400
Phisical activity level Ⅱ	2000	2050	1950	1850	1650
Phisical activity level Ⅲ	2300	2350	2250	2100	-
**Nutrients with EAR**					
Protein, g/d	40	40	40	40	40
Vitamin A, μg RAE/d	450	500	500	500	450
Thiamine, mg/d	0.9	0.9	0.9	0.9	0.8
Riboflavin, mg/d	1	1	1	1	0.9
Niacin, mg NE/d	9	10	9	9	9
Vitamin B-6, mg/d	1	1	1	1	1
Vitamin B-12, μg/d	2	2	2	2	2
Folate, μg/d	200	200	200	200	200
Vitamin C, mg/d	85	85	85	80	80
Sodium, mg/d	600	600	600	600	600
Calcium, mg/d	550	550	550	550	500
Magnesium, mg/d	230	240	240	230	220
Iron, mg/d (for non-menstruating females)	5.5	5.5	5.5	5	5
Iron, mg/d (for menstruating females)	8.5	9	9	-	-
Zinc, mg/d	7	7	7	7	6
**Nutrients with DG**					
Copper, mg/d	≥0.6	≥0.6	≥0.6	≥0.6	≥0.6
Protein, % energy	13–20	13–20	14–20	15–20	15–20
Fat, % energy	20–30	20–30	20–30	20–30	20–30
SFA, % energy	≤7	≤7	≤7	≤7	≤7
Carbohydrate, % energy	50–65	50–65	50–65	50–65	50–65
Dietary fibre, g/d	≥18	≥18	≥18	≥17	≥17
Sodium, g salt equivalent/d	<6.5	<6.5	<6.5	<6.5	<6.5
**Nutrients with AI**					
Potassium, mg/d	2600	2600	2600	2600	2600
n-6 PUFA, g/d	8	8	8	8	7
n-3 PUFA, g/d	1.6	1.6	1.9	2	1.8
Vitamin D, μg/d	8.5	8.5	8.5	8.5	8.5
Vitamin E, mg/d	5	5.5	6	6.5	6.5
Vitamin K, μg/d	150	150	150	150	150
Pantothenic acid, mg/d	5	5	5	5	5
Potassium, mg/d	2000	2000	2000	2000	2000
Phosphorus, mg/d	800	800	800	800	800
**Nutrients with UL**					
Manganese, mg/d	3.5	3.5	3.5	3.5	3.5
Vitamin A, μg RAE/d	2700	2700	2700	2700	2700
Vitamin D, μg/d	100	100	100	100	100
Vitamin E, mg/d	650	700	700	650	650
Niacin, mg NE/d [c]	60 (15)	250 (65)	250 (65)	250 (65)	250 (60)
Vitamin B-6, mg/d	45	45	45	40	40
Folate, μg/d	900	1000	1000	900	900
Calcium, mg/d	2500	2500	2500	2500	2500
Phosphorus, mg/d	3000	3000	3000	3000	3000
Iron, mg/d	40	40	40	40	40
Zinc, mg/d	35	35	35	35	30
Copper, mg/d	7	7	7	7	7
Manganese, mg/d	11	11	11	11	11

AI, Adequate Intake; DG, Tentative Dietary Goal for Preventing Lifestyle-related Diseases; EAR, Estimated Average Requirement; SD, standard deviation; SFA, saturated fatty acid; PUFA, polyunsaturated fatty acid. [a] Hyphens indicate the absence of a reference value. [b] The reference values for energy were not considered in this study. [c] The amount of nicotinamide. The values in parentheses are the amount of nicotinic acid.

Table S5. Usual intake distribution of energy and nutrients among Japanese aged 1–2 years [a].

	Males (N = 149)									Females (N = 142)								
Variables	5th	10th	25th	50th	75th	90th	95th	kurtosis	skewness	5th	10th	25th	50th	75th	90th	95th	kurtosis	skewness
**Energy, kcal/d**	905	965	1100	1197	1331	1514	1599	3.22	0.17	835	881	1004	1078	1197	1293	1385	2.96	0.07
**Nutrients without DRI values**																		
Fat, g/d	23.5	27.6	32.0	37.4	44.0	50.7	58.6	3.59	0.64	23.9	25.6	29.1	33.8	38.6	43.9	46.8	3.22	0.19
SFA, g/d	7.0	8.4	10.5	12.2	14.3	17.5	19.2	3.37	0.52	7.5	8.0	9.2	10.6	12.9	15.0	16.4	3.38	0.50
Carbohydrate, g/d	134.9	144.0	158.2	172.4	186.7	208.9	230.5	3.57	0.26	124.0	130.3	140.3	156.4	171.7	186.5	197.9	3.54	0.24
**Nutrients with EAR**																		
Protein, g/d	27.8	31.5	36.7	41.5	47.5	54.2	58.3	3.29	0.24	26.6	29.4	34.8	39.2	43.8	46.7	51.3	3.20	0.04
Vitamin A, μg RAE/d	199	234	299	361	454	520	578	3.45	0.41	218	255	287	340	440	533	573	3.16	0.59
Thiamine, mg/d	0.40	0.40	0.50	0.60	0.70	0.80	0.80	3.10	0.29	0.40	0.40	0.50	0.50	0.60	0.70	0.70	3.98	0.41
Riboflavin, mg/d	0.50	0.50	0.70	0.80	0.90	1.00	1.10	3.07	0.10	0.50	0.60	0.60	0.70	0.90	1.00	1.00	4.37	0.56
Niacin, mg NE/d	11.3	12.4	14.5	16.5	19.1	22.2	24.3	3.65	0.46	10.5	11.5	14.0	15.5	17.6	19.6	20.7	3.47	0.20
Vitamin B-6, mg/d	0.50	0.60	0.70	0.80	0.90	1.00	1.20	5.56	0.87	0.50	0.50	0.60	0.70	0.80	0.90	1.00	3.62	0.38
Vitamin B-12, μg/d	1.7	1.9	2.3	2.9	3.5	4.2	4.5	3.37	0.53	1.6	1.8	2.1	2.8	3.4	3.9	4.5	3.44	0.61
Folate, μg/d	104	121	141	168	205	233	246	2.66	0.15	105	116	143	170	197	219	248	3.22	0.31
Vitamin C, mg/d	34	39	50	61	73	87	94	3.16	0.34	32	38	48	59	75	86	98	3.22	0.51
Sodium, mg/d	1355	1540	1840	2112	2323	2639	2700	3.69	-0.28	1249	1400	1603	1988	2254	2530	2702	4.84	0.63
Calcium, mg/d	240	254	325	403	482	546	586	3.41	0.40	217	258	305	368	455	542	596	6.08	1.12
Magnesium, mg/d	103	115	129	147	169	188	197	2.71	0.06	98	106	125	141	155	173	186	4.82	0.66
Iron, mg/d	2.8	3.0	3.5	3.9	4.7	5.5	5.7	4.35	0.72	2.8	3.0	3.3	4.0	4.3	4.8	5.0	3.36	0.28
Zinc, mg/d	3.5	3.8	4.4	5.1	6.0	6.4	7.0	3.03	0.19	3.2	3.7	4.1	4.7	5.1	5.8	6.2	3.28	0.11
Copper, mg/d	0.40	0.50	0.60	0.60	0.70	0.80	0.90	3.38	0.46	0.40	0.50	0.50	0.60	0.70	0.70	0.80	3.50	0.11
**Nutrients with DG**																		
Protein, % energy	11.6	12.1	13.1	13.8	14.7	15.7	16.0	4.51	-0.63	12.2	12.4	13.5	14.6	15.2	15.8	16.5	3.33	-0.21
Fat, % energy	22.0	23.0	25.8	28.1	30.3	33.1	34.4	3.65	0.09	22.4	23.7	25.4	27.4	29.8	31.7	32.4	3.82	-0.30
SFA, % energy	6.4	7.1	8.1	9.2	10.2	11.1	11.6	3.03	-0.08	6.5	7.1	7.9	8.9	9.9	10.8	11.2	3.41	0.09
Carbohydrate, % energy	49.7	51.8	55.5	58.2	60.8	63.7	66.5	3.20	-0.03	51.5	53.0	55.3	58.0	60.2	62.0	64.6	3.92	0.36
Dietary fibre, g/d	7.4	8.4	9.6	10.6	12.9	14.4	15.4	3.54	0.43	7.0	7.6	9.1	10.6	11.7	13.2	14.2	3.51	0.29
Sodium, g salt equivalent/	3.4	3.9	4.7	5.4	5.9	6.7	6.9	3.69	-0.28	3.2	3.6	4.1	5.0	5.7	6.4	6.9	4.84	0.63
Potassium, mg/d	1052	1184	1361	1531	1781	1957	2086	3.49	0.17	1002	1109	1270	1470	1669	1825	1953	4.16	0.54
**Nutrients with AI**																		
n-6 PUFA, g/d	3.40	3.80	4.50	5.50	6.30	7.30	8.30	4.66	0.88	3.40	3.60	4.40	5.00	5.70	6.40	6.80	2.85	0.13
n-3 PUFA, g/d	0.50	0.60	0.70	0.90	1.10	1.30	1.50	3.05	0.57	0.50	0.60	0.70	0.90	1.00	1.30	1.30	3.16	0.55
Vitamin D, μg/d	1.7	2.1	2.8	3.5	4.7	5.5	6.1	3.10	0.62	2.0	2.3	2.7	3.6	4.3	5.3	5.6	2.56	0.32
Vitamin E, mg/d	2.8	3.3	3.9	4.4	5.2	5.7	6.3	9.09	1.40	2.6	2.8	3.5	4.1	4.7	5.2	5.7	2.91	0.18
Vitamin K, μg/d	50	70	94	120	156	201	235	4.32	0.97	65	72	97	129	163	196	222	6.04	1.24
Pantothenic acid, mg/d	2.60	2.80	3.40	3.80	4.50	5.10	5.30	2.80	0.11	2.60	2.80	3.30	3.60	4.10	4.50	4.90	3.73	0.31
Potassium, mg/d	1052	1184	1361	1531	1781	1957	2086	3.49	0.17	1002	1109	1270	1470	1669	1825	1953	4.16	0.54
Phosphorus, mg/d	409	495	565	669	758	876	907	2.83	0.06	428	460	554	624	702	786	814	3.95	0.41
Manganese, mg/d	1.10	1.20	1.40	1.60	1.80	2.20	2.40	3.31	0.62	1.00	1.10	1.30	1.50	1.70	1.80	2.10	3.89	0.51

AI, Adequate Intake; DG, Tentative Dietary Goal for Preventing Lifestyle-related Diseases; DRIs, Dietary Reference Intakes; EAR, Estimated Average Requirement; NE, niacin equivalent; SFA, saturated fatty acid; PUFA, polyunsaturated fatty acid; RAE, retinol active equivalent. [a] Usual intake was estimated using the Multiple Source Method based on 8-day weighed dietary-record data.

Table S6. Usual intake distribution of energy and nutrients among Japanese aged 3–5 years [a].

	Males (N = 225)									Females (N = 217)								
Variables	5th	10th	25th	50th	75th	90th	95th	kurtosis	skewness	5th	10th	25th	50th	75th	90th	95th	kurtosis	skewness
**Energy, kcal/d**	1149	1234	1343	1455	1597	1738	1808	3.17	0.34	1056	1087	1227	1367	1486	1602	1695	3.16	0.38
**Nutrients without DRI values**																		
Fat, g/d	37.8	39.9	44.6	49.7	55.2	61.4	63.7	2.88	0.36	32.3	33.8	39.5	46.2	51.6	57.6	62.1	3.01	0.34
SFA, g/d	11.7	12.3	13.8	15.9	17.8	20.3	21.2	3.59	0.55	9.4	10.5	12.4	14.4	16.4	19.1	20.1	3.45	0.42
Carbohydrate, g/d	155.2	170.7	183.8	200.9	219.4	236.3	250.4	3.41	0.31	149.1	155.1	171.4	184.7	202.1	224.9	232.5	3.30	0.47
**Nutrients with EAR**																		
Protein, g/d	40.0	41.7	45.9	52.0	57.5	65.1	68.5	3.20	0.58	35.4	37.2	42.1	48.0	53.4	59.2	61.5	2.76	0.14
Vitamin A, μg RAE/d	238	280	340	405	475	567	620	4.66	0.83	223	274	337	393	464	548	622	4.94	0.92
Thiamine, mg/d	0.60	0.60	0.70	0.70	0.80	0.90	1.00	3.03	0.34	0.50	0.50	0.60	0.70	0.80	0.90	0.90	3.26	0.33
Riboflavin, mg/d	0.70	0.70	0.80	0.90	1.10	1.30	1.40	5.95	1.26	0.60	0.60	0.70	0.90	1.00	1.10	1.20	3.60	0.49
Niacin, mg NE/d	16.0	16.8	18.3	21.1	23.7	25.7	27.5	2.76	0.39	14.3	15.1	16.8	19.8	22.3	24.5	25.5	2.92	0.27
Vitamin B-6, mg/d	0.60	0.70	0.80	0.90	1.00	1.10	1.20	3.14	0.59	0.60	0.60	0.70	0.90	1.00	1.10	1.10	3.46	0.28
Vitamin B-12, μg/d	2.2	2.6	3.0	3.6	4.4	5.3	5.8	3.15	0.65	2.0	2.3	2.8	3.4	4.0	4.7	5.4	3.83	0.68
Folate, μg/d	131	144	166	196	234	277	295	3.25	0.61	127	139	161	192	223	254	269	3.83	0.43
Vitamin C, mg/d	40	46	57	68	84	101	113	4.93	0.97	38	45	55	69	85	96	103	3.45	0.26
Sodium, mg/d	1742	1941	2214	2545	2903	3245	3451	3.84	0.50	1676	1802	2118	2433	2790	3209	3292	3.37	0.37
Calcium, mg/d	277	329	395	472	567	669	757	6.06	1.20	245	274	341	421	488	571	629	4.93	0.90
Magnesium, mg/d	128	135	151	171	196	230	241	3.27	0.67	114	127	141	163	182	201	211	3.45	0.31
Iron, mg/d	3.4	3.7	4.2	4.9	5.6	6.2	6.8	3.07	0.44	3.3	3.5	4.0	4.6	5.3	5.8	6.4	6.32	0.92
Zinc, mg/d	4.8	5.1	5.6	6.3	7.1	7.8	8.5	3.32	0.56	4.1	4.5	5.1	5.8	6.5	7.1	7.6	2.67	0.14
Copper, mg/d	0.60	0.60	0.70	0.70	0.90	1.00	1.00	3.10	0.49	0.50	0.60	0.60	0.70	0.80	0.90	0.90	3.15	0.42
**Nutrients with DG**																		
Protein, % energy	12.6	13.0	13.5	14.3	14.9	15.7	15.9	2.71	0.07	12.3	12.7	13.5	14.2	14.9	15.6	16.0	3.16	-0.11
Fat, % energy	25.9	27.0	28.8	30.1	31.7	33.3	34.6	2.85	-0.07	24.3	25.8	28.0	30.0	32.2	33.9	34.9	2.69	-0.14
SFA, % energy	7.5	7.9	8.9	9.7	10.5	11.3	11.8	2.68	-0.09	7.2	7.8	8.7	9.6	10.3	11.2	11.7	3.80	0.01
Carbohydrate, % energy	51.0	51.7	53.6	55.4	57.1	59.0	60.6	2.99	0.16	50.0	51.3	53.4	55.4	58.1	61.1	62.3	2.94	0.31
Dietary fibre, g/d	9.4	10.3	11.6	13.4	14.8	16.5	17.9	3.59	0.58	9.1	9.7	10.9	12.5	14.1	16.0	16.9	4.28	0.31
Sodium, g salt equivalent	4.4	4.9	5.6	6.5	7.4	8.2	8.8	3.84	0.50	4.3	4.6	5.4	6.2	7.1	8.2	8.4	3.37	0.37
Potassium, mg/d	1356	1438	1571	1795	2044	2275	2549	3.86	0.74	1177	1300	1450	1703	1925	2116	2257	3.46	0.35
**Nutrients with AI**																		
n-6 PUFA, g/d	5.00	5.40	6.20	7.10	8.20	9.10	9.60	3.25	0.36	4.60	5.00	5.70	6.60	7.50	8.40	9.00	2.71	0.13
n-3 PUFA, g/d	0.80	0.90	1.10	1.30	1.50	1.70	1.80	3.38	0.60	0.70	0.80	0.90	1.20	1.40	1.50	1.60	3.47	0.19
Vitamin D, μg/d	2.2	2.5	3.0	4.0	5.1	6.1	6.7	4.19	0.77	2.2	2.6	3.2	4.2	5.4	6.2	7.2	6.39	1.10
Vitamin E, mg/d	4.0	4.2	4.6	5.3	6.1	6.7	7.1	5.58	0.80	3.5	3.9	4.4	5.1	6.0	6.5	7.0	2.63	0.18
Vitamin K, μg/d	84	92	110	140	187	232	277	5.77	1.41	67	78	104	130	164	203	251	4.98	1.11
Pantothenic acid, mg/d	3.50	3.60	4.10	4.60	5.20	5.80	6.30	5.00	1.04	3.00	3.30	3.80	4.20	4.80	5.30	5.80	3.19	0.36
Potassium, mg/d	1356	1438	1571	1795	2044	2275	2549	3.86	0.74	1177	1300	1450	1703	1925	2116	2257	3.46	0.35
Phosphorus, mg/d	586	648	717	805	901	1008	1073	4.31	0.81	525	554	659	746	826	912	991	3.07	0.25
Manganese, mg/d	1.30	1.40	1.70	1.90	2.20	2.50	2.70	3.13	0.50	1.30	1.40	1.60	1.80	2.10	2.30	2.60	3.13	0.47

AI, Adequate Intake; DG, Tentative Dietary Goal for Preventing Lifestyle-related Diseases; DRIs, Dietary Reference Intakes; EAR, Estimated Average Requirement; NE, niacin equivalent; SFA, saturated fatty acid; PUFA, polyunsaturated fatty acid; RAE, retinol active equivalent. [a] Usual intake was estimated using the Multiple Source Method based on 8-day weighed dietary-record data.

Table S7. Usual intake distribution of energy and nutrients among Japanese aged 6–7 years [a].

	Males (N = 98)									Females (N = 108)								
Variables	5th	10th	25th	50th	75th	90th	95th	kurtosis	skewness	5th	10th	25th	50th	75th	90th	95th	kurtosis	skewness
**Energy, kcal/d**	1408	1482	1612	1712	1835	1947	1982	3.11	0.02	1274	1329	1437	1574	1739	1877	1920	3.05	0.30
**Nutrients without DRI values**																		
Fat, g/d	45.1	49.0	53.0	58.1	64.8	70.6	77.5	3.35	0.21	41.3	44.1	49.2	54.0	63.2	67.8	72.0	2.86	0.43
SFA, g/d	14.1	15.7	17.1	19.1	21.1	23.7	24.5	3.27	-0.04	13.5	13.9	15.6	17.6	20.8	22.9	25.2	3.34	0.65
Carbohydrate, g/d	194.4	200.6	214.2	235.8	251.5	266.1	273.1	2.74	0.03	175.1	180.4	195.8	212.4	233.6	254.4	260.8	2.78	0.36
**Nutrients with EAR**																		
Protein, g/d	48.8	51.3	56.2	61.0	65.5	71.6	75.5	3.43	0.03	42.9	45.5	50.8	57.1	63.7	70.1	71.4	2.50	0.04
Vitamin A, μg RAE/d	267	299	378	428	495	547	623	3.04	-0.05	326	356	393	450	505	577	654	4.99	0.77
Thiamine, mg/d	0.70	0.70	0.80	0.90	1.00	1.10	1.10	3.00	0.40	0.60	0.70	0.70	0.80	0.90	1.00	1.10	3.22	0.51
Riboflavin, mg/d	0.80	0.80	1.00	1.10	1.20	1.30	1.40	3.08	0.37	0.80	0.80	0.90	1.00	1.20	1.30	1.40	2.88	0.35
Niacin, mg NE/d	19.0	19.8	22.9	24.8	27.7	31.2	32.3	3.15	0.08	16.5	17.9	20.4	23.2	25.9	28.7	29.8	2.96	0.28
Vitamin B-6, mg/d	0.80	0.80	0.90	1.00	1.10	1.30	1.40	3.35	0.17	0.70	0.70	0.80	0.90	1.10	1.20	1.20	2.64	0.25
Vitamin B-12, μg/d	2.9	3.1	3.5	4.2	5.0	6.1	6.6	2.68	0.64	2.6	3.0	3.4	4.2	5.1	5.9	6.3	9.00	1.59
Folate, μg/d	153	166	189	220	249	272	298	3.58	0.46	147	175	196	218	251	286	304	4.28	0.64
Vitamin C, mg/d	36	46	57	71	86	101	108	2.90	0.30	45	48	59	71	87	101	121	3.68	0.78
Sodium, mg/d	2237	2485	2654	2992	3507	3727	4099	2.93	0.51	2212	2340	2630	2925	3257	3552	3814	3.97	0.40
Calcium, mg/d	339	388	447	517	599	704	757	3.42	0.48	329	370	422	509	593	697	748	3.15	0.60
Magnesium, mg/d	143	157	172	196	216	239	252	3.40	0.30	135	140	161	194	209	232	250	3.19	0.29
Iron, mg/d	4.0	4.4	4.9	5.5	6.2	7.2	7.7	2.90	0.48	4.1	4.4	5.0	5.5	6.1	6.8	7.5	3.52	0.46
Zinc, mg/d	6.0	6.3	6.8	7.4	8.1	8.8	9.2	2.91	0.26	5.1	5.5	6.1	7.0	7.7	8.3	9.0	2.66	0.20
Copper, mg/d	0.60	0.70	0.80	0.90	1.00	1.10	1.10	2.49	0.23	0.60	0.70	0.70	0.80	0.90	1.00	1.10	2.98	0.18
**Nutrients with DG**																		
Protein, % energy	12.4	12.8	13.3	14.3	14.9	15.7	16.4	2.85	0.15	12.8	13.1	13.7	14.5	15.1	15.8	16.1	3.46	0.36
Fat, % energy	24.4	26.5	28.7	31.3	33.2	34.2	35.1	2.77	-0.44	27.6	27.9	29.5	31.3	32.7	34.5	35.7	3.18	0.28
SFA, % energy	7.8	8.4	9.2	10.0	11.0	11.9	12.4	3.04	-0.11	8.2	8.8	9.3	10.1	10.9	12.1	12.5	2.71	0.37
Carbohydrate, % energy	50.4	51.0	52.5	54.4	56.7	59.9	61.2	2.88	0.46	48.0	49.4	52.0	54.1	56.6	58.8	59.5	2.73	-0.15
Dietary fibre, g/d	11.6	11.8	13.4	15.1	17.1	18.3	18.7	3.29	0.32	11.4	12.0	13.0	15.0	16.2	17.8	18.8	3.32	0.29
Sodium, g salt equivalent	5.7	6.3	6.7	7.6	8.9	9.5	10.4	2.93	0.51	5.6	5.9	6.7	7.4	8.3	9.0	9.7	3.97	0.40
Potassium, mg/d	1540	1582	1767	2014	2227	2388	2556	2.75	0.00	1425	1469	1710	1943	2129	2389	2571	3.57	0.44
**Nutrients with AI**																		
n-6 PUFA, g/d	6.30	6.80	7.60	8.20	9.20	10.10	11.30	4.91	0.88	5.70	6.20	6.90	8.00	8.70	10.10	10.40	2.91	0.37
n-3 PUFA, g/d	0.90	1.00	1.10	1.40	1.70	1.90	2.10	3.65	0.68	0.80	0.90	1.10	1.30	1.50	1.80	2.00	2.76	0.39
Vitamin D, μg/d	2.8	3.6	4.0	5.0	5.9	6.7	7.6	2.73	0.22	2.9	3.2	3.8	4.6	5.4	6.5	7.0	2.95	0.50
Vitamin E, mg/d	4.5	4.8	5.5	6.0	6.6	7.1	7.9	6.44	0.91	4.1	4.3	4.9	5.7	6.5	7.3	7.6	2.28	0.19
Vitamin K, μg/d	102	107	114	143	173	221	263	6.91	1.56	87	99	126	152	186	226	238	3.68	0.64
Pantothenic acid, mg/d	4.10	4.30	4.70	5.20	5.70	6.20	6.40	3.61	0.22	3.60	3.90	4.30	4.90	5.60	6.30	6.60	2.76	0.26
Potassium, mg/d	1540	1582	1767	2014	2227	2388	2556	2.75	0.00	1425	1469	1710	1943	2129	2389	2571	3.57	0.44
Phosphorus, mg/d	716	780	852	930	1014	1118	1146	3.45	-0.01	637	689	762	887	988	1073	1124	2.80	0.16
Manganese, mg/d	1.70	1.80	2.10	2.20	2.60	2.80	2.90	2.37	0.13	1.60	1.70	1.90	2.20	2.40	2.80	3.00	3.53	0.59

AI, Adequate Intake; DG, Tentative Dietary Goal for Preventing Lifestyle-related Diseases; DRIs, Dietary Reference Intakes; EAR, Estimated Average Requirement; NE, niacin equivalent; SFA, saturated fatty acid; PUFA, polyunsaturated fatty acid; RAE, retinol active equivalent. [a] Usual intake was estimated using the Multiple Source Method based on 8-day weighed dietary-record data.

Table S8. Usual intake distribution of energy and nutrients among Japanese aged 8–9 years [a].

	Males (N = 63)									Females (N = 72)								
Variables	5th	10th	25th	50th	75th	90th	95th	kurtosis	skewness	5th	10th	25th	50th	75th	90th	95th	kurtosis	skewness
**Energy, kcal/d**	1651	1683	1774	1975	2176	2343	2551	3.51	0.73	1424	1501	1645	1785	1895	2030	2146	6.64	1.30
**Nutrients without DRI values**																		
Fat, g/d	51.4	53.1	59.4	69.8	79.1	89.8	94.7	2.75	0.56	49.2	51.8	55.5	59.6	68.3	77.1	82.7	5.28	1.22
SFA, g/d	16.7	17.5	19.5	22.0	25.2	29.5	30.0	2.48	0.39	15.0	16.1	17.0	18.9	22.2	25.1	27.5	4.77	0.90
Carbohydrate, g/d	220.8	224.7	237.3	260.9	292.1	319.4	326.3	3.76	0.79	192.1	206.5	219.5	241.3	259.4	282.8	320.6	4.88	0.93
**Nutrients with EAR**																		
Protein, g/d	51.2	55.5	63.8	70.2	79.5	85.9	90.8	3.44	0.35	48.0	53.4	57.8	62.7	68.7	76.6	81.0	6.07	1.21
Vitamin A, μg RAE/d	369	386	418	502	586	666	740	2.74	0.52	335	361	405	485	565	613	691	3.74	0.70
Thiamine, mg/d	0.80	0.80	0.90	1.00	1.20	1.30	1.40	2.68	0.40	0.70	0.80	0.80	0.90	1.00	1.10	1.20	4.18	0.85
Riboflavin, mg/d	0.90	1.00	1.20	1.30	1.40	1.50	1.60	3.22	0.15	0.90	0.90	1.00	1.20	1.30	1.40	1.50	4.14	0.66
Niacin, mg NE/d	20.7	22.7	25.2	28.9	32.8	37.0	38.5	4.19	0.54	19.1	20.7	23.5	25.7	28.3	32.5	35.8	5.15	1.09
Vitamin B-6, mg/d	0.90	0.90	1.00	1.20	1.30	1.50	1.60	7.06	1.39	0.70	0.80	0.90	1.00	1.20	1.30	1.50	3.87	0.71
Vitamin B-12, μg/d	3.4	3.6	4.2	5.3	6.5	7.7	9.0	3.53	0.83	2.8	3.1	3.6	4.4	5.5	6.6	7.4	7.08	1.47
Folate, μg/d	200	213	233	265	288	302	325	2.87	0.25	151	177	207	244	282	333	374	3.24	0.61
Vitamin C, mg/d	57	63	70	78	90	99	106	2.71	0.11	43	49	63	79	96	117	140	6.07	1.35
Sodium, mg/d	2756	2915	3237	3622	3953	4442	4640	2.94	0.28	2648	2842	3179	3396	3695	4043	4354	5.75	1.04
Calcium, mg/d	467	480	535	599	720	792	831	2.84	0.52	372	419	481	544	617	662	735	4.79	0.81
Magnesium, mg/d	183	188	196	234	252	270	286	2.38	0.22	152	166	187	210	236	263	291	3.23	0.55
Iron, mg/d	4.8	5.1	5.7	6.6	7.4	8.2	8.7	2.62	0.27	4.4	4.7	5.4	6.1	7.0	7.6	8.4	3.79	0.81
Zinc, mg/d	6.7	7.1	7.8	9.0	9.9	10.5	11.5	2.47	0.23	5.7	6.2	6.8	7.7	8.5	9.2	9.8	4.05	0.74
Copper, mg/d	0.80	0.80	0.90	1.00	1.10	1.20	1.20	2.74	0.26	0.70	0.80	0.90	0.90	1.10	1.10	1.30	3.64	0.54
**Nutrients with DG**																		
Protein, % energy	12.4	12.7	13.7	14.4	15.2	15.9	16.6	3.14	0.16	12.2	12.9	13.5	14.4	14.9	15.8	16.7	2.97	0.00
Fat, % energy	27.4	29.0	30.2	31.5	32.8	34.3	34.8	3.01	-0.12	26.9	27.6	29.1	31.0	33.0	33.9	34.5	2.23	-0.19
SFA, % energy	8.8	9.2	9.8	10.3	10.6	11.1	11.2	3.10	-0.55	8.0	8.2	8.9	10.0	10.9	11.3	11.5	3.13	-0.53
Carbohydrate, % energy	49.5	50.1	52.1	54.0	55.4	57.6	59.6	3.01	0.43	49.6	50.9	52.2	54.6	56.9	58.0	59.2	2.42	-0.01
Dietary fibre, g/d	13.6	15.1	16.2	18.0	19.9	21.2	22.4	2.55	0.05	12.4	12.9	15.2	16.7	19.0	20.7	22.8	2.77	0.20
Sodium, g salt equivalent	7.0	7.4	8.2	9.2	10.0	11.3	11.8	2.94	0.28	6.7	7.2	8.1	8.6	9.4	10.3	11.1	5.75	1.04
Potassium, mg/d	1884	1915	2062	2338	2554	2830	2881	2.59	0.43	1487	1719	1871	2174	2410	2515	2857	3.42	0.44
**Nutrients with AI**																		
n-6 PUFA, g/d	6.80	7.00	7.90	9.90	11.80	13.10	13.90	3.08	0.64	7.00	7.30	7.90	8.80	10.20	11.00	12.10	2.96	0.62
n-3 PUFA, g/d	1.00	1.00	1.40	1.70	2.00	2.30	2.50	3.02	0.58	1.10	1.20	1.40	1.50	1.80	2.00	2.10	2.25	0.18
Vitamin D, μg/d	2.9	3.2	4.1	5.1	6.8	7.7	8.5	3.40	0.58	2.4	2.9	3.5	4.9	6.2	7.4	8.2	3.10	0.62
Vitamin E, mg/d	5.6	5.7	6.1	6.8	7.6	8.2	9.2	3.47	0.64	5.1	5.2	5.8	6.5	7.2	8.0	8.5	2.95	0.30
Vitamin K, μg/d	134	145	161	176	204	224	230	2.58	0.44	102	107	126	166	195	239	279	6.62	1.41
Pantothenic acid, mg/d	4.80	5.00	5.40	5.90	6.60	7.60	7.90	2.82	0.52	4.30	4.40	4.90	5.50	6.00	6.60	6.90	3.52	0.60
Potassium, mg/d	1884	1915	2062	2338	2554	2830	2881	2.59	0.43	1487	1719	1871	2174	2410	2515	2857	3.42	0.44
Phosphorus, mg/d	826	854	977	1070	1197	1347	1361	2.74	0.28	755	805	867	961	1037	1123	1285	5.08	1.16
Manganese, mg/d	2.00	2.10	2.40	2.70	3.00	3.30	3.60	2.98	0.48	1.70	1.90	2.20	2.40	3.00	3.60	4.00	4.53	1.08

AI, Adequate Intake; DG, Tentative Dietary Goal for Preventing Lifestyle-related Diseases; DRIs, Dietary Reference Intakes; EAR, Estimated Average Requirement; NE, niacin equivalent; SFA, saturated fatty acid; PUFA, polyunsaturated fatty acid; RAE, retinol active equivalent. [a] Usual intake was estimated using the Multiple Source Method based on 8-day weighed dietary-record data.

Table S9. Usual intake distribution of energy and nutrients among Japanese aged 10–11 years [a].

	Males (N = 77)									Females (N = 67)								
Variables	5th	10th	25th	50th	75th	90th	95th	kurtosis	skewness	5th	10th	25th	50th	75th	90th	95th	kurtosis	skewness
**Energy, kcal/d**	1710	1787	1944	2071	2284	2526	2622	5.66	1.04	1579	1626	1768	1914	2074	2249	2310	4.72	0.75
**Nutrients without DRI values**																		
Fat, g/d	56.1	60.2	66.5	73.3	84.6	95.7	102.7	3.51	0.51	50.1	53.7	62.3	67.8	75.8	82.8	85.6	5.41	0.72
SFA, g/d	16.6	17.6	20.7	23.3	26.8	29.8	32.0	4.88	0.80	15.0	16.5	18.8	21.5	25.0	29.1	29.6	2.34	0.16
Carbohydrate, g/d	233.0	240.2	256.9	277.9	295.8	318.7	355.7	6.26	1.34	213.2	219.6	234.5	255.8	276.2	296.9	305.4	2.58	0.27
**Nutrients with EAR**																		
Protein, g/d	57.1	60.4	69.5	76.4	87.3	97.2	104.6	4.37	0.82	58.8	60.3	63.2	68.4	73.5	79.7	85.3	4.94	0.92
Vitamin A, μg RAE/d	369	383	450	576	658	796	840	8.12	1.67	353	406	457	536	634	701	719	2.33	0.03
Thiamine, mg/d	0.90	0.90	1.00	1.10	1.20	1.40	1.40	3.89	0.69	0.80	0.90	0.90	1.00	1.10	1.20	1.20	3.14	-0.11
Riboflavin, mg/d	0.90	1.00	1.10	1.30	1.50	1.90	2.20	4.28	1.12	1.00	1.00	1.10	1.20	1.40	1.60	1.70	2.98	0.42
Niacin, mg NE/d	23.6	24.9	28.6	31.2	36.1	41.1	43.4	4.64	0.98	23.5	24.1	25.9	28.4	30.9	33.2	37.0	4.16	0.91
Vitamin B-6, mg/d	1.00	1.00	1.10	1.30	1.50	1.70	1.80	9.80	1.78	0.90	0.90	1.10	1.20	1.30	1.50	1.60	3.03	0.47
Vitamin B-12, μg/d	3.1	3.5	4.6	5.4	7.4	8.9	9.5	4.94	1.22	3.4	3.5	4.0	4.7	5.5	6.6	7.1	3.07	0.79
Folate, μg/d	183	199	228	287	318	358	422	9.04	1.77	199	222	241	274	295	320	333	3.76	0.33
Vitamin C, mg/d	51	59	70	90	107	127	134	12.99	2.23	57	62	75	90	105	114	119	3.14	0.41
Sodium, mg/d	2937	3151	3549	3923	4269	4840	5065	4.99	0.82	2853	2940	3189	3583	3864	4194	4439	3.27	0.49
Calcium, mg/d	396	419	511	613	755	967	1065	5.80	1.39	396	429	511	585	690	843	969	3.38	0.76
Magnesium, mg/d	184	195	214	243	274	308	355	9.02	1.78	178	186	206	228	247	263	288	5.55	1.07
Iron, mg/d	5.3	5.6	6.3	6.9	7.8	8.9	10.1	6.02	1.35	5.2	5.3	6.0	6.6	7.2	8.1	8.6	3.78	0.63
Zinc, mg/d	6.8	7.6	8.4	9.4	10.8	12.0	12.9	4.23	0.75	7.0	7.1	7.8	8.3	8.8	9.6	9.8	3.41	0.53
Copper, mg/d	0.90	0.90	1.00	1.10	1.20	1.40	1.40	5.37	1.22	0.80	0.90	0.90	1.00	1.10	1.10	1.20	4.67	0.77
**Nutrients with DG**																		
Protein, % energy	13.1	13.5	14.0	14.8	15.5	16.3	16.8	3.18	0.03	12.7	13.1	13.7	14.5	15.2	16.3	16.4	2.78	0.19
Fat, % energy	28.3	28.9	30.2	31.4	33.0	34.0	35.0	2.97	-0.03	27.5	28.8	30.3	31.8	32.9	33.7	34.4	4.14	-0.70
SFA, % energy	8.4	8.7	9.4	9.9	10.6	11.2	11.5	3.14	0.31	8.2	8.5	9.2	10.0	10.9	11.7	12.7	3.02	0.23
Carbohydrate, % energy	49.2	50.1	51.4	53.3	55.2	57.1	58.6	2.83	0.01	50.1	51.0	51.8	53.8	54.7	57.9	59.3	4.49	1.00
Dietary fibre, g/d	14.1	16.0	17.5	18.9	20.6	22.6	24.6	6.53	1.18	15.1	15.7	16.9	17.9	19.6	20.7	22.9	4.26	0.67
Sodium, g salt equivalent	7.5	8.0	9.0	10.0	10.8	12.3	12.9	4.99	0.82	7.2	7.5	8.1	9.1	9.8	10.7	11.3	3.27	0.49
Potassium, mg/d	1752	1886	2236	2436	2813	3245	3523	9.79	1.81	1771	1814	2119	2349	2546	2814	2952	3.65	0.54
**Nutrients with AI**																		
n-6 PUFA, g/d	7.70	8.70	9.80	10.60	12.20	13.90	15.90	3.84	0.66	7.50	7.80	8.50	9.70	11.20	11.70	12.80	10.44	1.96
n-3 PUFA, g/d	1.10	1.30	1.60	1.90	2.10	2.50	3.00	3.97	0.67	1.00	1.20	1.30	1.50	1.90	2.30	2.40	2.87	0.63
Vitamin D, μg/d	3.1	3.9	4.7	6.2	8.0	9.4	10.9	3.45	0.64	3.3	3.5	4.6	5.5	7.1	8.8	9.6	6.09	1.48
Vitamin E, mg/d	5.0	5.6	6.6	7.2	8.2	10.3	11.1	4.98	1.21	5.1	5.5	6.1	6.9	7.9	8.7	8.9	4.63	0.87
Vitamin K, μg/d	107	126	154	184	225	278	303	5.67	1.23	124	138	160	182	209	239	261	3.05	0.37
Pantothenic acid, mg/d	4.60	5.00	5.70	6.40	7.30	8.60	9.70	4.66	0.96	4.80	5.20	5.50	5.90	6.60	7.00	7.30	3.98	0.52
Potassium, mg/d	1752	1886	2236	2436	2813	3245	3523	9.79	1.81	1771	1814	2119	2349	2546	2814	2952	3.65	0.54
Phosphorus, mg/d	817	866	1017	1149	1278	1567	1676	6.14	1.29	838	863	962	1040	1146	1241	1335	4.62	0.90
Manganese, mg/d	2.10	2.30	2.60	2.90	3.10	3.60	3.80	12.30	2.09	2.20	2.30	2.50	2.80	3.10	3.40	3.70	3.61	0.52

AI, Adequate Intake; DG, Tentative Dietary Goal for Preventing Lifestyle-related Diseases; DRIs, Dietary Reference Intakes; EAR, Estimated Average Requirement; NE, niacin equivalent; SFA, saturated fatty acid; PUFA, polyunsaturated fatty acid; RAE, retinol active equivalent. [a] Usual intake was estimated using the Multiple Source Method based on 8-day weighed dietary-record data.

Table S10. Usual intake distribution of energy and nutrients among Japanese aged 12–14 years [a].

	Males (N = 107)									Females (N = 95)								
Variables	5th	10th	25th	50th	75th	90th	95th	kurtosis	skewness	5th	10th	25th	50th	75th	90th	95th	kurtosis	skewness
**Energy, kcal/d**	1888	2063	2397	2680	3009	3326	3565	4.94	0.71	1536	1734	1916	2072	2257	2476	2586	3.23	0.17
**Nutrients without DRI values**																		
Fat, g/d	65.5	69.5	79.8	92.4	103.7	117.0	125.4	6.00	1.07	55.9	58.2	66.5	74.5	82.5	94.7	100.6	3.38	0.60
SFA, g/d	19.3	20.8	25.4	29.9	33.4	36.4	39.7	4.76	0.63	17.3	18.2	20.7	24.0	27.3	30.3	33.7	4.00	0.75
Carbohydrate, g/d	249.3	274.5	323.3	364.2	431.5	474.2	510.8	3.36	0.50	201.0	217.2	244.7	278.1	305.1	318.1	352.0	2.88	-0.06
**Nutrients with EAR**																		
Protein, g/d	63.0	70.5	85.7	95.8	104.3	113.5	123.8	4.77	0.62	56.3	62.1	69.2	75.1	82.7	90.8	95.8	2.87	-0.03
Vitamin A, µg RAE/d	434	460	541	613	749	865	941	4.24	0.73	354	373	449	544	618	722	810	2.87	0.38
Thiamine, mg/d	1.00	1.00	1.20	1.40	1.60	1.80	1.90	3.05	0.40	0.80	0.80	0.90	1.10	1.20	1.40	1.40	4.06	0.50
Riboflavin, mg/d	1.00	1.10	1.40	1.60	1.80	2.00	2.30	3.58	0.63	0.90	1.00	1.10	1.30	1.40	1.60	1.70	2.53	0.14
Niacin, mg NE/d	26.0	28.1	35.2	39.1	43.2	48.9	55.5	4.98	0.76	23.0	24.7	28.2	31.2	34.8	38.8	40.8	2.82	0.07
Vitamin B-6, mg/d	1.00	1.10	1.40	1.60	1.80	2.10	2.40	4.30	0.86	0.90	1.00	1.10	1.20	1.40	1.60	1.60	2.50	0.10
Vitamin B-12, µg/d	4.0	4.3	5.1	5.7	7.0	8.1	9.4	3.81	0.83	3.2	3.5	4.4	5.2	6.4	7.5	8.0	2.83	0.42
Folate, µg/d	211	223	262	319	388	437	524	4.38	1.12	195	205	244	289	326	379	424	3.28	0.57
Vitamin C, mg/d	63	66	78	99	134	162	179	4.62	1.18	42	54	74	91	112	134	157	3.83	0.75
Sodium, mg/d	3441	3621	3965	4570	4985	5793	6428	3.87	0.79	2816	3082	3344	3801	4270	4619	4862	2.74	0.08
Calcium, mg/d	417	480	568	689	876	993	1059	3.08	0.50	374	401	456	561	661	773	824	2.59	0.45
Magnesium, mg/d	192	215	247	293	330	372	426	7.02	1.29	170	190	211	236	263	296	303	2.35	0.12
Iron, mg/d	5.9	6.4	7.4	8.4	9.7	10.9	12.1	5.78	1.16	5.5	5.7	6.5	7.1	8.0	9.0	9.5	2.95	0.29
Zinc, mg/d	8.1	9.2	10.5	12.0	13.3	15.6	16.0	4.38	0.64	7.0	7.4	8.3	9.1	10.0	10.7	11.3	3.96	0.31
Copper, mg/d	0.90	1.00	1.20	1.30	1.60	1.80	1.90	7.33	1.40	0.80	0.90	1.00	1.10	1.20	1.30	1.40	2.71	-0.02
**Nutrients with DG**																		
Protein, % energy	12.3	12.6	13.4	14.0	14.9	15.7	15.9	2.84	-0.04	12.4	13.4	14.0	14.7	15.3	16.0	16.6	3.44	-0.31
Fat, % energy	26.5	27.1	28.5	30.6	32.1	34.0	35.0	2.97	0.46	27.4	28.4	29.9	32.3	34.1	36.2	37.2	2.47	0.00
SFA, % energy	8.1	8.3	8.9	9.6	10.3	10.7	11.2	3.05	0.14	8.4	9.0	9.5	10.2	11.0	11.9	12.3	2.93	0.22
Carbohydrate, % energy	49.2	50.9	53.2	55.5	58.0	59.3	60.1	2.75	-0.40	47.0	49.0	50.5	52.6	55.4	57.4	58.1	2.60	0.03
Dietary fibre, g/d	16.1	17.1	20.2	23.7	26.5	30.3	33.6	4.51	0.94	13.6	15.2	17.3	19.1	21.1	22.4	23.7	3.46	-0.14
Sodium, g salt equivalent	8.7	9.2	10.1	11.6	12.7	14.7	16.3	3.87	0.79	7.2	7.8	8.5	9.7	10.8	11.7	12.3	2.74	0.08
Potassium, mg/d	1952	2127	2440	2926	3380	3773	4479	4.36	0.92	1665	1934	2141	2449	2677	3013	3115	2.73	-0.09
**Nutrients with AI**																		
n-6 PUFA, g/d	9.20	9.90	11.90	13.10	15.20	17.10	18.40	5.68	1.05	7.80	8.60	9.70	10.60	12.10	13.20	14.10	3.37	0.30
n-3 PUFA, g/d	1.50	1.60	1.80	2.00	2.50	2.70	2.90	3.01	0.38	1.30	1.40	1.50	1.80	2.10	2.30	2.40	2.99	0.50
Vitamin D, µg/d	3.7	4.0	5.3	6.2	7.9	9.5	10.2	3.96	0.76	3.5	4.0	4.8	5.8	7.5	9.4	10.2	3.48	0.80
Vitamin E, mg/d	6.0	6.8	8.1	9.1	10.4	11.9	13.0	4.49	0.75	5.5	6.0	6.7	7.6	8.8	9.6	10.5	2.99	0.48
Vitamin K, µg/d	122	140	170	213	270	357	437	4.75	1.24	115	138	164	190	251	289	303	2.41	0.38
Pantothenic acid, mg/d	5.00	5.70	6.80	7.80	8.80	10.10	10.90	4.54	0.75	4.30	5.00	5.70	6.10	6.80	7.50	7.90	3.31	0.15
Potassium, mg/d	1952	2127	2440	2926	3380	3773	4479	4.36	0.92	1665	1934	2141	2449	2677	3013	3115	2.73	-0.09
Phosphorus, mg/d	918	998	1195	1393	1515	1750	1908	4.20	0.59	767	886	971	1092	1193	1316	1435	2.73	0.14
Manganese, mg/d	2.60	2.80	3.10	3.60	4.30	4.90	5.60	4.77	1.11	2.10	2.40	2.60	3.00	3.40	4.20	4.50	4.26	0.96

AI, Adequate Intake; DG, Tentative Dietary Goal for Preventing Lifestyle-related Diseases; DRIs, Dietary Reference Intakes; EAR, Estimated Average Requirement; NE, niacin equivalent; SFA, saturated fatty acid; PUFA, polyunsaturated fatty acid; RAE, retinol active equivalent. [a] Usual intake was estimated using the Multiple Source Method based on 8-day weighed dietary-record data.

Table S11. Usual intake distribution of energy and nutrients among Japanese aged 15–17 years [a].

	Males (N = 122)									Females (N = 106)								
Variables	5th	10th	25th	50th	75th	90th	95th	kurtosis	skewness	5th	10th	25th	50th	75th	90th	95th	kurtosis	skewness
**Energy, kcal/d**	1918	2054	2423	2947	3310	3818	4314	3.48	0.61	1423	1549	1701	1926	2206	2434	2501	2.48	0.07
**Nutrients without DRI values**																		
Fat, g/d	63.5	66.9	80.1	103.5	117.5	132.1	141.4	2.59	0.20	49.1	52.8	61.2	71.8	81.8	90.2	97.6	2.73	0.23
SFA, g/d	18.7	20.5	24.6	30.8	36.5	41.5	42.6	2.36	0.08	14.4	15.7	18.6	21.8	24.9	28.6	29.6	2.30	0.03
Carbohydrate, g/d	257.1	281.2	336.3	396.6	457.0	534.4	672.4	4.14	1.04	180.0	197.1	227.2	251.2	282.8	318.1	334.7	2.98	0.16
**Nutrients with EAR**																		
Protein, g/d	62.7	68.3	87.0	104.6	119.9	140.7	149.2	4.31	0.70	51.8	54.3	61.6	70.4	79.5	89.8	93.0	2.92	0.28
Vitamin A, μg RAE/d	325	358	491	649	782	936	1097	2.98	0.51	285	319	373	489	558	725	779	3.50	0.70
Thiamine, mg/d	0.80	1.00	1.30	1.50	1.70	2.10	2.30	3.83	0.53	0.70	0.80	0.90	1.00	1.10	1.30	1.40	3.89	0.61
Riboflavin, mg/d	1.00	1.10	1.30	1.60	1.90	2.30	2.40	2.91	0.39	0.80	0.90	1.00	1.10	1.30	1.50	1.60	3.56	0.52
Niacin, mg NE/d	25.4	29.4	36.6	43.9	51.0	57.6	64.2	5.82	1.09	20.9	23.2	25.6	29.8	34.6	37.8	40.0	3.12	0.36
Vitamin B-6, mg/d	0.90	1.00	1.40	1.70	2.00	2.20	2.50	5.32	0.89	0.80	0.80	1.00	1.20	1.40	1.60	1.90	3.92	0.67
Vitamin B-12, μg/d	3.3	3.9	4.9	6.3	7.8	9.8	11.5	4.83	1.02	2.3	2.8	3.5	4.7	5.5	7.5	9.0	4.85	1.17
Folate, μg/d	191	232	289	361	427	496	544	6.56	1.01	177	195	226	276	327	430	459	5.60	1.25
Vitamin C, mg/d	49	59	86	113	139	158	174	7.87	1.03	46	52	68	84	110	139	175	6.20	1.44
Sodium, mg/d	3039	3513	4109	4746	5648	6817	7582	3.51	0.70	2658	2861	3258	3659	4278	4691	4844	2.95	0.27
Calcium, mg/d	310	358	475	604	789	922	1139	3.83	0.90	265	296	374	461	564	618	686	2.66	0.20
Magnesium, mg/d	170	203	253	304	345	404	446	4.06	0.57	152	161	184	216	246	295	315	3.26	0.56
Iron, mg/d	5.5	6.5	7.7	9.0	10.7	12.2	13.7	4.31	0.78	4.8	5.2	6.0	6.7	7.9	9.1	9.5	3.75	0.64
Zinc, mg/d	7.7	8.6	11.1	13.3	15.0	17.3	18.9	4.87	0.60	6.0	6.7	7.5	8.7	9.5	10.7	11.3	2.87	0.14
Copper, mg/d	0.90	1.00	1.20	1.50	1.70	2.00	2.30	4.66	0.96	0.70	0.80	0.90	1.00	1.10	1.30	1.40	3.20	0.42
**Nutrients with DG**																		
Protein, % energy	12.3	12.6	13.1	14.1	15.0	15.8	16.2	5.80	0.93	12.4	12.7	13.5	14.4	15.5	16.8	17.4	2.72	0.27
Fat, % energy	24.3	25.1	28.3	30.2	32.6	34.9	36.0	2.93	-0.12	27.6	28.2	30.6	32.7	34.6	36.1	37.1	3.89	0.24
SFA, % energy	6.9	7.6	8.3	9.1	10.2	11.1	11.4	2.81	0.04	7.9	8.3	9.1	9.8	10.6	11.3	11.7	2.86	0.14
Carbohydrate, % energy	48.8	49.8	52.2	55.0	57.8	61.0	62.9	3.54	-0.06	47.1	47.9	49.8	53.0	54.9	57.9	59.4	4.55	-0.34
Dietary fibre, g/d	13.7	16.8	21.0	25.0	28.7	33.5	37.1	3.58	0.44	11.7	13.3	15.3	17.6	20.0	23.1	24.5	4.78	0.79
Sodium, g salt equivalent	7.7	8.9	10.4	12.1	14.3	17.3	19.3	3.51	0.70	6.8	7.3	8.3	9.3	10.9	11.9	12.3	2.95	0.27
Potassium, mg/d	1578	1975	2530	3056	3563	4007	4084	4.47	0.45	1447	1531	1838	2124	2532	2967	3324	3.51	0.70
**Nutrients with AI**																		
n-6 PUFA, g/d	8.50	9.50	11.70	14.40	16.80	19.50	21.10	2.55	0.28	7.40	8.20	9.10	10.30	12.10	13.10	13.90	6.06	1.08
n-3 PUFA, g/d	1.30	1.40	1.70	2.20	3.00	3.40	3.80	2.79	0.53	1.10	1.20	1.50	1.70	2.10	2.70	2.80	4.62	0.95
Vitamin D, μg/d	4.4	4.9	5.6	7.5	9.9	12.5	13.6	2.69	0.48	2.9	3.3	4.6	5.8	7.5	10.5	11.3	2.89	0.74
Vitamin E, mg/d	5.7	6.4	8.2	9.9	11.8	13.6	14.7	2.73	0.23	5.4	5.8	6.5	7.4	8.5	10.1	10.7	3.04	0.39
Vitamin K, μg/d	122	151	191	246	319	411	447	3.93	0.86	107	123	153	198	246	308	352	4.94	1.18
Pantothenic acid, mg/d	4.70	5.20	7.20	8.30	9.50	10.90	11.90	3.52	0.39	4.00	4.30	4.90	5.60	6.30	7.00	7.60	2.97	0.31
Potassium, mg/d	1578	1975	2530	3056	3563	4007	4084	4.47	0.45	1447	1531	1838	2124	2532	2967	3324	3.51	0.70
Phosphorus, mg/d	850	947	1209	1445	1657	1930	2018	3.06	0.38	699	749	859	995	1109	1263	1378	2.95	0.32
Manganese, mg/d	2.80	3.20	3.60	4.30	5.20	6.30	7.30	5.36	1.33	2.10	2.30	2.60	3.00	3.40	4.10	4.40	21.14	3.08

AI, Adequate Intake; DG, Tentative Dietary Goal for Preventing Lifestyle-related Diseases; DRIs, Dietary Reference Intakes; EAR, Estimated Average Requirement; NE, niacin equivalent; SFA, saturated fatty acid; PUFA, polyunsaturated fatty acid; RAE, retinol active equivalent. [a] Usual intake was estimated using the Multiple Source Method based on 8-day weighed dietary-record data.

Table S12. Usual intake distribution of energy and nutrients among Japanese aged 18–29 years [a].

Variables	Males (N = 271)									Females (N = 291)								
	5th	10th	25th	50th	75th	90th	95th	kurtosis	skewness	5th	10th	25th	50th	75th	90th	95th	kurtosis	skewness
**Energy, kcal/d**	1475	1601	1819	2169	2519	2855	3097	3.82	0.42	1175	1278	1484	1687	1867	2000	2108	3.24	0.06
**Nutrients without DRI values**																		
Fat, g/d	42.0	49.6	62.1	74.1	87.0	103.1	112.2	3.63	0.41	39.2	42.4	49.6	57.4	67.4	75.7	84.0	3.65	0.38
SFA, g/d	12.1	14.0	17.2	21.1	26.1	30.6	34.9	3.71	0.61	10.6	12.1	14.3	17.3	20.1	23.6	25.7	3.43	0.40
Carbohydrate, g/d	191.6	212.8	246.6	292.6	337.0	392.7	424.2	4.21	0.51	152.5	169.3	195.8	221.7	244.9	270.4	291.7	3.62	-0.09
**Nutrients with EAR**																		
Protein, g/d	48.2	52.9	63.8	77.0	91.6	105.6	114.3	3.80	0.52	42.5	47.6	53.4	60.6	69.0	76.3	82.2	3.00	0.17
Vitamin A, μg RAE/d	220	244	317	422	545	661	791	3.65	0.75	227	261	320	424	521	610	690	6.47	1.26
Thiamine, mg/d	0.70	0.80	0.90	1.10	1.30	1.60	1.70	3.67	0.58	0.60	0.70	0.80	0.90	1.00	1.10	1.20	3.85	0.48
Riboflavin, mg/d	0.80	0.80	1.00	1.20	1.50	1.70	1.90	3.37	0.61	0.70	0.80	0.90	1.00	1.20	1.40	1.50	3.01	0.35
Niacin, mg NE/d	20.2	22.8	27.4	34.0	40.5	46.7	51.8	4.03	0.57	17.6	20.2	22.6	26.1	30.1	34.3	36.3	3.75	0.50
Vitamin B-6, mg/d	0.70	0.80	1.00	1.30	1.60	1.90	2.00	3.41	0.49	0.60	0.70	0.90	1.00	1.20	1.40	1.50	3.27	0.52
Vitamin B-12, μg/d	2.2	2.5	3.4	4.5	6.1	8.0	9.5	4.34	1.04	2.0	2.5	3.0	3.9	4.9	6.2	7.1	6.03	1.30
Folate, μg/d	138	177	224	282	344	428	491	5.61	1.04	154	174	205	255	315	369	427	4.62	0.97
Vitamin C, mg/d	32	44	60	80	106	136	164	4.16	0.97	37	46	58	79	99	119	141	5.10	1.08
Sodium, mg/d	2562	2979	3509	4062	4731	5316	6030	3.54	0.33	2272	2516	2921	3312	3821	4253	4501	3.10	0.16
Calcium, mg/d	230	259	324	414	541	683	783	3.93	0.98	240	257	320	381	472	573	633	6.76	1.22
Magnesium, mg/d	139	161	196	238	279	312	360	4.33	0.66	131	148	167	198	232	272	306	6.00	1.10
Iron, mg/d	4.6	5.0	6.1	7.4	8.6	10.0	11.0	3.93	0.54	4.3	4.6	5.3	6.2	7.3	8.5	9.2	4.36	0.78
Zinc, mg/d	5.8	6.6	7.7	9.3	11.2	12.8	14.4	3.93	0.56	4.9	5.6	6.2	7.2	8.2	9.0	9.7	3.10	0.21
Copper, mg/d	0.70	0.80	0.90	1.10	1.30	1.60	1.70	5.18	0.85	0.60	0.70	0.80	0.90	1.00	1.20	1.30	3.92	0.70
**Nutrients with DG**																		
Protein, % energy	11.6	12.3	12.9	14.1	15.3	16.5	17.6	6.47	1.05	12.2	12.7	13.6	14.6	15.8	16.9	17.9	13.55	1.96
Fat, % energy	23.6	25.2	27.1	30.1	32.4	35.3	36.8	4.57	0.38	24.5	26.2	28.5	30.9	33.1	35.8	37.4	3.78	0.00
SFA, % energy	6.1	7.0	7.7	8.7	9.8	10.8	11.5	3.42	0.23	6.7	7.3	8.2	9.1	10.1	11.1	11.6	3.12	0.06
Carbohydrate, % energy	45.3	47.9	51.8	54.7	57.8	61.3	63.1	4.51	-0.44	45.3	46.6	50.6	53.8	56.8	59.3	60.6	4.82	-0.35
Dietary fibre, g/d	12.2	13.5	15.8	19.1	22.5	26.2	28.0	4.35	0.55	10.9	12.1	13.8	16.2	18.2	20.4	22.2	3.18	0.36
Sodium, g salt equivalent	6.5	7.6	8.9	10.3	12.0	13.5	15.3	3.54	0.33	5.8	6.4	7.4	8.4	9.7	10.8	11.4	3.10	0.16
Potassium, mg/d	1290	1505	1822	2260	2782	3256	3534	3.48	0.48	1269	1432	1638	1930	2292	2658	2951	4.15	0.77
**Nutrients with AI**																		
n-6 PUFA, g/d	6.70	7.30	9.30	11.20	13.10	14.90	17.10	4.20	0.57	5.80	6.40	7.40	8.80	10.20	12.20	13.00	2.93	0.45
n-3 PUFA, g/d	0.90	1.10	1.40	1.90	2.40	2.70	3.10	3.80	0.51	0.70	0.80	1.10	1.50	1.90	2.30	2.60	4.15	0.84
Vitamin D, μg/d	2.0	2.6	3.6	4.9	6.9	9.0	10.3	6.25	1.28	2.4	2.8	3.7	5.0	6.5	8.8	9.8	8.72	1.68
Vitamin E, mg/d	4.3	5.0	6.3	7.8	9.3	10.8	12.0	3.28	0.38	4.0	4.7	5.6	6.5	7.6	8.9	9.4	3.57	0.43
Vitamin K, μg/d	88	105	148	202	257	335	407	5.98	1.24	91	107	141	180	237	287	319	5.36	1.09
Pantothenic acid, mg/d	3.60	4.10	4.80	5.90	7.10	8.20	9.00	3.82	0.56	3.30	3.70	4.10	4.90	5.60	6.40	6.90	2.88	0.29
Potassium, mg/d	1290	1505	1822	2260	2782	3256	3534	3.48	0.48	1269	1432	1638	1930	2292	2658	2951	4.15	0.77
Phosphorus, mg/d	630	693	851	1037	1217	1413	1562	3.38	0.47	600	649	735	834	980	1089	1194	3.14	0.39
Manganese, mg/d	2.20	2.40	2.90	3.60	4.40	5.30	6.50	21.71	2.94	1.80	1.90	2.40	2.80	3.40	4.10	4.60	21.64	2.85

AI, Adequate Intake; DG, Tentative Dietary Goal for Preventing Lifestyle-related Diseases; DRIs, Dietary Reference Intakes; EAR, Estimated Average Requirement; NE, niacin equivalent; SFA, saturated fatty acid; PUFA, polyunsaturated fatty acid; RAE, retinol active equivalent. [a] Usual intake was estimated using the Multiple Source Method based on 8-day weighed dietary-record data.

Table S13. Usual intake distribution of energy and nutrients among Japanese aged 30–49 years [a].

	Males (N = 439)									Females (N = 443)								
Variables	5th	10th	25th	50th	75th	90th	95th	kurtosis	skewness	5th	10th	25th	50th	75th	90th	95th	kurtosis	skewness
**Energy, kcal/d**	1537	1621	1811	2098	2409	2730	2998	4.27	0.68	1266	1399	1577	1772	1976	2203	2304	6.75	0.73
**Nutrients without DRI values**																		
Fat, g/d	43.2	49.2	56.7	68.7	81.7	95.4	107.2	3.98	0.66	40.2	44.8	51.3	59.6	69.1	77.8	82.2	5.11	0.56
SFA, g/d	11.4	12.9	15.7	19.6	24.0	28.2	32.2	3.68	0.69	11.0	12.7	15.0	17.8	21.0	24.5	26.6	3.88	0.56
Carbohydrate, g/d	185.3	206.9	235.9	276.4	316.0	364.3	383.9	3.77	0.57	161.1	177.0	201.2	234.5	260.9	289.4	301.3	4.25	0.36
**Nutrients with EAR**																		
Protein, g/d	48.6	54.1	64.4	75.1	87.0	98.5	103.1	5.57	0.71	46.2	50.1	56.9	64.5	72.7	79.0	83.8	4.06	0.33
Vitamin A, μg RAE/d	213	257	349	449	589	722	823	4.08	0.81	258	297	383	482	573	696	817	5.57	1.09
Thiamine, mg/d	0.70	0.80	0.90	1.10	1.30	1.40	1.50	3.67	0.46	0.60	0.70	0.80	0.90	1.10	1.20	1.30	3.60	0.24
Riboflavin, mg/d	0.80	0.90	1.00	1.20	1.50	1.70	1.80	3.82	0.60	0.70	0.80	1.00	1.10	1.30	1.50	1.60	3.91	0.51
Niacin, mg NE/d	21.6	24.1	28.5	33.8	39.7	45.7	49.6	4.09	0.61	20.4	22.4	24.8	28.4	32.7	36.5	39.6	3.93	0.56
Vitamin B-6, mg/d	0.80	0.80	1.00	1.30	1.50	1.80	2.00	4.66	0.82	0.70	0.80	1.00	1.10	1.30	1.50	1.60	4.68	0.70
Vitamin B-12, μg/d	2.5	3.0	4.0	5.0	6.5	8.4	9.5	5.76	1.24	2.1	2.6	3.3	4.4	5.6	7.2	9.2	5.02	1.24
Folate, μg/d	165	189	245	297	360	452	489	4.00	0.77	177	204	249	297	352	425	473	15.39	2.02
Vitamin C, mg/d	38	46	64	82	108	133	156	4.56	0.94	48	56	72	92	116	140	161	4.24	0.84
Sodium, mg/d	2684	3039	3581	4075	4612	5225	5652	3.45	0.09	2458	2648	3081	3473	3942	4399	4616	4.28	0.50
Calcium, mg/d	253	285	344	424	551	695	772	3.25	0.78	271	305	367	447	533	631	727	4.41	0.88
Magnesium, mg/d	156	175	211	254	292	346	380	4.12	0.68	159	177	200	232	263	300	332	7.42	1.26
Iron, mg/d	4.6	5.2	6.3	7.4	8.7	10.3	11.4	5.73	0.89	4.6	5.0	6.0	7.0	7.9	9.0	9.8	6.56	1.03
Zinc, mg/d	5.8	6.5	7.6	9.0	10.5	11.8	12.7	7.14	0.89	5.1	5.8	6.6	7.6	8.5	9.4	9.8	3.26	0.09
Copper, mg/d	0.70	0.80	0.90	1.10	1.30	1.50	1.70	3.78	0.57	0.70	0.70	0.90	1.00	1.10	1.30	1.40	7.76	1.13
**Nutrients with DG**																		
Protein, % energy	11.3	12.0	13.1	14.2	15.2	16.4	17.4	7.89	0.89	12.3	12.9	13.7	14.6	15.6	16.6	17.2	6.85	0.68
Fat, % energy	21.1	23.0	26.0	29.2	32.4	34.7	36.0	3.21	0.03	24.2	25.7	27.9	30.4	32.5	34.3	35.5	3.64	-0.20
SFA, % energy	5.7	6.1	7.1	8.3	9.4	10.4	11.0	3.49	0.32	6.5	7.0	7.9	9.0	9.9	10.8	11.5	3.54	0.16
Carbohydrate, % energy	41.6	44.9	49.2	53.5	57.1	60.0	62.4	3.65	-0.32	44.7	47.0	50.3	53.2	56.2	58.9	60.5	4.97	-0.74
Dietary fibre, g/d	12.4	13.7	16.6	19.3	22.7	25.9	27.8	3.40	0.30	12.1	13.5	15.3	17.8	20.0	21.6	24.2	7.11	1.02
Sodium, g salt equivalent	6.8	7.7	9.1	10.4	11.7	13.3	14.4	3.45	0.09	6.2	6.7	7.8	8.8	10.0	11.2	11.7	4.28	0.50
Potassium, mg/d	1479	1580	1917	2347	2804	3199	3624	3.70	0.57	1509	1663	1949	2260	2632	2914	3186	5.39	0.77
**Nutrients with AI**																		
n-6 PUFA, g/d	6.40	7.40	8.90	10.80	12.90	15.30	16.80	3.67	0.56	6.40	7.10	8.20	9.50	11.00	12.30	13.60	4.96	0.77
n-3 PUFA, g/d	1.00	1.20	1.60	2.00	2.40	3.00	3.30	3.19	0.44	0.90	1.10	1.30	1.70	2.10	2.50	2.80	9.31	1.67
Vitamin D, μg/d	2.4	3.0	3.9	5.6	7.8	10.2	11.5	5.86	1.21	2.3	2.7	3.8	5.3	7.0	8.9	9.9	4.83	1.06
Vitamin E, mg/d	4.7	5.2	6.2	7.6	9.0	10.7	11.8	4.11	0.76	4.7	5.2	6.1	7.0	8.3	9.6	10.1	5.41	0.88
Vitamin K, μg/d	103	120	156	205	263	340	393	7.69	1.40	121	137	163	200	249	321	377	7.27	1.63
Pantothenic acid, mg/d	3.80	4.00	4.90	5.80	6.80	7.60	8.50	3.58	0.47	3.60	4.00	4.60	5.20	6.00	6.60	7.10	4.39	0.58
Potassium, mg/d	1479	1580	1917	2347	2804	3199	3624	3.70	0.57	1509	1663	1949	2260	2632	2914	3186	5.39	0.77
Phosphorus, mg/d	657	737	869	1020	1205	1390	1475	3.60	0.49	649	709	808	922	1059	1157	1262	4.41	0.51
Manganese, mg/d	2.30	2.50	3.00	3.50	4.30	5.30	6.00	7.68	1.36	2.00	2.30	2.60	3.10	3.70	4.60	5.40	158.55	10.03

AI, Adequate Intake; DG, Tentative Dietary Goal for Preventing Lifestyle-related Diseases; DRIs, Dietary Reference Intakes; EAR, Estimated Average Requirement; NE, niacin equivalent; SFA, saturated fatty acid; PUFA, polyunsaturated fatty acid; RAE, retinol active equivalent. [a] Usual intake was estimated using the Multiple Source Method based on 8-day weighed dietary-record data.

Table S14. Usual intake distribution of energy and nutrients among Japanese aged 50–64 years [a].

	Males (N = 336)									Females (N = 362)								
Variables	5th	10th	25th	50th	75th	90th	95th	kurtosis	skewness	5th	10th	25th	50th	75th	90th	95th	kurtosis	skewness
**Energy, kcal/d**	1591	1779	1996	2266	2527	2775	2999	4.87	0.43	1408	1513	1675	1867	2049	2234	2395	3.20	0.20
**Nutrients without DRI values**																		
Fat, g/d	44.6	51.1	60.7	70.2	82.9	94.3	101.1	3.96	0.47	45.5	48.9	55.4	62.9	71.9	79.9	85.6	3.74	0.52
SFA, g/d	11.9	13.2	15.9	19.7	23.1	27.2	30.4	3.87	0.67	12.2	13.6	15.9	18.5	21.0	24.1	26.3	3.36	0.43
Carbohydrate, g/d	198.4	218.1	248.1	285.3	322.0	360.9	385.5	3.42	0.15	171.6	186.2	212.9	241.2	273.5	299.4	309.4	2.94	0.06
**Nutrients with EAR**																		
Protein, g/d	57.0	61.6	72.7	83.5	94.1	105.9	111.8	5.29	0.57	53.6	56.8	61.8	70.6	78.4	86.2	92.1	3.21	0.37
Vitamin A, μg RAE/d	290	342	421	533	669	837	923	7.13	1.40	324	370	464	565	686	790	858	4.48	0.69
Thiamine, mg/d	0.80	0.80	1.00	1.10	1.40	1.50	1.70	3.44	0.48	0.70	0.80	0.90	1.00	1.10	1.30	1.40	3.47	0.46
Riboflavin, mg/d	0.90	1.00	1.20	1.40	1.60	1.80	2.00	3.77	0.67	0.90	1.00	1.10	1.30	1.50	1.70	1.80	3.35	0.40
Niacin, mg NE/d	25.6	28.5	32.5	38.1	44.2	50.6	53.9	5.20	0.76	23.8	24.8	28.2	31.8	35.9	40.4	43.7	3.37	0.54
Vitamin B-6, mg/d	0.90	1.00	1.20	1.50	1.80	2.10	2.30	3.61	0.56	0.90	0.90	1.10	1.30	1.50	1.80	1.90	3.15	0.45
Vitamin B-12, μg/d	3.7	4.2	5.6	7.2	8.8	11.5	13.2	5.76	1.24	3.6	3.9	4.5	5.6	7.3	8.6	9.7	6.08	1.12
Folate, μg/d	216	237	288	358	449	505	563	3.52	0.58	234	261	305	360	427	501	541	5.22	0.90
Vitamin C, mg/d	52	61	76	104	139	175	193	3.69	0.75	67	76	96	119	151	181	204	3.17	0.59
Sodium, mg/d	2868	3236	3817	4474	5060	5755	6302	3.71	0.40	2793	2970	3400	3808	4414	4782	5069	2.94	0.29
Calcium, mg/d	306	343	423	514	644	767	824	4.16	0.81	341	373	444	527	620	711	772	3.67	0.57
Magnesium, mg/d	194	217	253	300	353	402	427	3.52	0.43	189	203	236	269	312	352	380	3.36	0.53
Iron, mg/d	5.8	6.3	7.2	8.7	10.1	11.6	12.2	5.01	0.68	5.4	6.2	7.0	7.9	9.1	10.2	10.8	4.04	0.57
Zinc, mg/d	6.3	7.0	8.1	9.4	10.9	12.2	13.0	4.59	0.39	5.8	6.3	7.1	8.0	8.9	10.0	10.5	3.47	0.42
Copper, mg/d	0.80	0.90	1.10	1.30	1.50	1.70	1.80	3.87	0.41	0.80	0.90	1.00	1.10	1.30	1.40	1.50	3.65	0.45
**Nutrients with DG**																		
Protein, % energy	12.2	12.7	13.7	14.8	15.9	16.9	17.3	3.42	0.05	13.0	13.6	14.3	15.2	16.3	17.2	17.9	5.20	0.75
Fat, % energy	21.1	22.7	25.1	28.1	30.9	33.3	34.9	2.94	-0.04	25.3	26.4	28.1	30.2	32.6	34.7	36.5	3.27	0.26
SFA, % energy	5.2	5.8	6.7	7.7	8.8	9.8	10.5	3.78	0.41	6.8	7.3	7.9	8.7	9.6	10.5	11.0	4.47	0.55
Carbohydrate, % energy	39.9	42.0	46.8	51.6	55.7	59.4	60.9	3.22	-0.39	43.8	45.3	49.1	52.6	55.9	58.4	60.0	3.57	-0.48
Dietary fibre, g/d	13.7	15.3	18.1	21.9	25.1	28.4	30.9	4.84	0.52	13.9	15.0	17.6	20.1	22.8	25.1	27.2	3.16	0.30
Sodium, g salt equivalent	7.3	8.2	9.7	11.4	12.9	14.6	16.0	3.71	0.40	7.1	7.5	8.6	9.7	11.2	12.1	12.9	2.94	0.29
Potassium, mg/d	1750	1982	2286	2783	3317	3769	4123	2.92	0.32	1766	1950	2265	2652	3083	3529	3767	3.22	0.39
**Nutrients with AI**																		
n-6 PUFA, g/d	7.40	8.20	9.60	11.60	13.50	15.50	17.00	4.73	0.61	7.30	7.70	8.90	10.30	11.50	13.30	14.20	3.31	0.51
n-3 PUFA, g/d	1.40	1.50	1.90	2.40	2.90	3.60	3.90	3.75	0.65	1.30	1.40	1.60	2.00	2.40	2.90	3.20	4.83	0.95
Vitamin D, μg/d	3.9	4.5	6.0	7.9	10.4	13.5	15.3	4.08	0.91	4.1	4.7	5.5	7.1	8.7	11.1	12.4	3.95	0.94
Vitamin E, mg/d	5.2	6.0	6.9	8.6	10.0	11.3	12.4	3.13	0.35	5.4	6.1	7.1	8.3	9.3	10.7	11.6	3.13	0.34
Vitamin K, μg/d	118	139	175	246	325	412	451	3.72	0.84	133	152	186	237	298	379	409	5.62	1.15
Pantothenic acid, mg/d	4.40	4.70	5.40	6.40	7.60	8.30	9.00	3.15	0.32	4.20	4.40	5.00	5.80	6.60	7.40	7.90	2.97	0.29
Potassium, mg/d	1750	1982	2286	2783	3317	3769	4123	2.92	0.32	1766	1950	2265	2652	3083	3529	3767	3.22	0.39
Phosphorus, mg/d	803	862	999	1170	1352	1521	1620	4.33	0.55	764	808	901	1043	1157	1295	1353	3.16	0.34
Manganese, mg/d	2.50	2.80	3.40	4.00	4.90	6.00	6.50	4.21	0.91	2.30	2.60	3.10	3.70	4.50	5.10	5.50	25.36	3.53

AI, Adequate Intake; DG, Tentative Dietary Goal for Preventing Lifestyle-related Diseases; DRIs, Dietary Reference Intakes; EAR, Estimated Average Requirement; NE, niacin equivalent; SFA, saturated fatty acid; PUFA, polyunsaturated fatty acid; RAE, retinol active equivalent. [a] Usual intake was estimated using the Multiple Source Method based on 8-day weighed dietary-record data.

Table S15. Usual intake distribution of energy and nutrients among Japanese aged 65–74 years [a].

	Males (N = 229)									Females (N = 243)								
Variables	5th	10th	25th	50th	75th	90th	95th	kurtosis	skewness	5th	10th	25th	50th	75th	90th	95th	kurtosis	skewness
**Energy, kcal/d**	1644	1774	2004	2215	2440	2681	2805	4.17	0.33	1510	1583	1709	1868	2049	2254	2373	12.11	1.71
**Nutrients without DRI values**																		
Fat, g/d	43.9	48.8	59.2	68.7	78.1	87.3	95.2	4.36	0.65	43.7	47.5	53.7	63.3	71.1	80.7	87.6	3.93	0.62
SFA, g/d	11.2	12.9	15.6	18.5	22.3	25.0	27.3	3.71	0.64	11.2	12.4	14.2	17.4	20.5	23.5	25.6	3.39	0.50
Carbohydrate, g/d	195.0	218.8	250.3	282.8	318.8	348.1	372.8	3.47	0.24	188.7	202.0	221.4	246.6	273.9	302.3	322.8	13.42	1.74
**Nutrients with EAR**																		
Protein, g/d	61.7	66.3	75.5	85.0	94.0	102.6	107.3	4.50	0.38	56.0	60.5	67.0	74.4	84.7	94.6	98.1	4.36	0.78
Vitamin A, μg RAE/d	353	391	509	628	770	953	1055	6.91	1.34	373	427	518	636	795	1028	1223	11.74	1.98
Thiamine, mg/d	0.70	0.80	1.00	1.10	1.30	1.50	1.70	3.71	0.61	0.70	0.80	0.90	1.10	1.20	1.40	1.50	4.14	0.70
Riboflavin, mg/d	1.00	1.10	1.30	1.50	1.70	1.90	2.10	4.56	0.68	1.00	1.00	1.20	1.40	1.70	1.90	2.10	3.08	0.55
Niacin, mg NE/d	27.6	29.2	33.0	38.0	43.4	48.3	51.3	4.98	0.68	24.2	26.5	29.7	33.8	38.5	43.1	46.2	4.29	0.83
Vitamin B-6, mg/d	1.10	1.20	1.40	1.60	1.80	2.20	2.30	4.24	0.67	1.00	1.10	1.30	1.50	1.70	2.00	2.10	3.67	0.69
Vitamin B-12, μg/d	4.6	5.3	6.4	8.0	10.3	12.9	14.5	5.80	1.29	3.6	4.0	5.5	7.1	9.2	12.2	13.8	4.64	1.17
Folate, μg/d	267	292	366	427	498	599	674	4.77	0.94	245	272	348	415	504	604	693	3.43	0.78
Vitamin C, mg/d	74	84	111	137	161	199	224	3.28	0.53	76	85	116	146	185	217	240	3.89	0.69
Sodium, mg/d	3094	3396	3905	4636	5291	5944	6406	3.18	0.13	3074	3146	3537	3977	4666	5427	5891	3.95	0.86
Calcium, mg/d	361	397	472	598	714	828	914	3.06	0.51	359	396	483	605	742	905	946	2.74	0.43
Magnesium, mg/d	220	239	279	318	363	417	449	5.46	0.91	206	224	260	296	349	413	458	4.20	0.91
Iron, mg/d	6.7	7.2	8.2	9.4	10.6	12.4	13.5	4.96	0.83	5.9	6.8	7.7	8.9	10.6	12.0	13.4	4.35	0.84
Zinc, mg/d	7.0	7.4	8.3	9.3	10.6	11.7	12.7	3.59	0.35	6.4	6.8	7.6	8.4	9.7	10.8	11.4	7.41	1.47
Copper, mg/d	0.90	1.00	1.20	1.30	1.50	1.70	1.80	3.96	0.51	0.90	0.90	1.10	1.20	1.40	1.60	1.80	4.16	0.90
**Nutrients with DG**																		
Protein, % energy	13.2	13.7	14.3	15.3	16.1	17.2	17.9	3.52	0.56	13.8	14.1	15.1	16.1	17.2	18.1	19.0	3.21	0.34
Fat, % energy	20.8	22.2	24.7	27.7	29.7	33.1	34.7	3.02	0.25	23.8	24.6	26.8	29.4	32.2	35.0	36.1	2.93	0.24
SFA, % energy	5.2	5.8	6.6	7.4	8.5	9.6	10.2	2.71	0.23	5.9	6.5	7.3	8.2	9.3	10.3	10.9	2.86	0.19
Carbohydrate, % energy	41.5	43.1	46.9	52.5	56.5	59.7	61.7	2.62	-0.17	44.2	46.6	50.0	53.5	56.2	59.4	60.4	3.04	-0.43
Dietary fibre, g/d	16.3	17.9	20.5	23.5	27.3	31.2	33.9	3.07	0.47	15.3	16.6	19.0	22.2	25.5	30.0	33.1	4.50	0.91
Sodium, g salt equivalent	7.9	8.6	9.9	11.8	13.4	15.1	16.3	3.18	0.13	7.8	8.0	9.0	10.1	11.9	13.8	15.0	3.95	0.86
Potassium, mg/d	1995	2250	2590	3012	3543	4175	4534	3.38	0.62	1923	2245	2606	2963	3488	4093	4586	4.27	0.87
**Nutrients with AI**																		
n-6 PUFA, g/d	6.70	7.70	9.10	11.00	12.80	14.50	15.30	6.10	0.97	6.60	7.30	8.60	10.10	11.80	13.60	14.70	3.00	0.45
n-3 PUFA, g/d	1.60	1.80	2.10	2.60	3.30	4.00	4.50	4.71	1.08	1.40	1.60	1.90	2.30	2.80	3.40	3.70	5.65	1.04
Vitamin D, μg/d	5.2	5.9	7.5	9.5	11.8	14.4	16.2	2.92	0.55	4.5	5.1	6.8	8.7	10.8	13.2	16.0	4.47	1.01
Vitamin E, mg/d	5.6	6.1	7.7	8.7	10.3	11.9	13.4	4.56	0.90	5.7	6.1	7.4	8.7	10.4	11.9	13.5	3.06	0.58
Vitamin K, μg/d	141	163	228	295	394	503	542	3.51	0.80	136	167	211	285	380	468	554	4.73	1.11
Pantothenic acid, mg/d	4.80	5.10	5.90	6.70	7.60	8.70	9.00	4.12	0.67	4.20	4.80	5.60	6.30	7.30	8.10	8.90	4.57	0.82
Potassium, mg/d	1995	2250	2590	3012	3543	4175	4534	3.38	0.62	1923	2245	2606	2963	3488	4093	4586	4.27	0.87
Phosphorus, mg/d	868	923	1095	1208	1354	1526	1632	4.80	0.56	790	860	975	1117	1284	1459	1547	3.67	0.64
Manganese, mg/d	2.70	3.10	3.70	4.40	5.30	6.20	6.90	27.19	3.36	2.70	2.80	3.40	3.90	4.90	6.00	6.60	6.17	1.42

AI, Adequate Intake; DG, Tentative Dietary Goal for Preventing Lifestyle-related Diseases; DRIs, Dietary Reference Intakes; EAR, Estimated Average Requirement; NE, niacin equivalent; SFA, saturated fatty acid; PUFA, polyunsaturated fatty acid; RAE, retinol active equivalent. [a] Usual intake was estimated using the Multiple Source Method based on 8-day weighed dietary-record data.

Table S16. Usual intake distribution of energy and nutrients among Japanese aged 75–79 years [a].

	Males (N = 100)									Females (N = 88)								
Variables	5th	10th	25th	50th	75th	90th	95th	kurtosis	skewness	5th	10th	25th	50th	75th	90th	95th	kurtosis	skewness
**Energy, kcal/d**	1675	1758	1941	2254	2486	2706	2927	4.46	0.71	1352	1387	1562	1785	2019	2153	2345	3.35	0.44
**Nutrients without DRI values**																		
Fat, g/d	41.7	46.5	54.5	65.3	75.9	88.6	103.4	8.92	1.69	34.1	37.3	46.6	55.3	65.5	72.8	77.6	2.66	0.06
SFA, g/d	11.5	12.5	14.0	17.7	20.9	25.7	29.3	5.78	1.29	9.0	9.8	12.5	15.3	18.6	21.5	22.5	2.69	0.17
Carbohydrate, g/d	221.3	232.4	247.4	295.9	329.8	365.2	391.2	3.31	0.63	183.9	196.2	217.7	245.8	277.8	307.0	326.6	3.72	0.69
**Nutrients with EAR**																		
Protein, g/d	61.2	63.7	73.7	84.9	96.1	110.2	127.1	3.86	0.69	48.7	53.4	60.8	71.3	81.3	88.9	94.6	3.05	0.26
Vitamin A, μg RAE/d	375	403	501	611	733	905	1094	5.14	1.27	363	405	514	636	763	940	1036	5.30	1.02
Thiamine, mg/d	0.80	0.90	0.90	1.10	1.30	1.50	1.80	3.92	0.98	0.60	0.70	0.80	0.90	1.10	1.30	1.40	3.23	0.63
Riboflavin, mg/d	1.10	1.10	1.30	1.50	1.70	2.00	2.30	4.31	0.99	0.90	0.90	1.10	1.30	1.60	1.80	2.00	3.45	0.41
Niacin, mg NE/d	26.2	27.0	32.5	38.1	44.6	50.3	60.1	4.76	0.99	20.9	23.6	26.4	31.0	35.8	39.0	42.8	2.87	0.04
Vitamin B-6, mg/d	1.00	1.10	1.40	1.60	1.90	2.40	2.80	4.52	1.01	0.80	0.90	1.10	1.30	1.60	1.80	1.90	2.81	0.31
Vitamin B-12, μg/d	3.9	4.6	6.5	9.0	11.3	14.1	16.1	4.09	0.87	3.9	4.1	5.3	6.8	8.2	10.3	11.6	5.44	1.34
Folate, μg/d	265	295	336	408	501	639	736	3.69	0.85	234	256	315	398	457	568	637	4.10	0.84
Vitamin C, mg/d	66	83	102	134	178	219	261	4.17	0.94	72	84	107	135	158	204	213	6.12	1.33
Sodium, mg/d	3472	3598	3972	4433	5080	5791	6210	9.40	1.75	2681	2803	3474	3954	4635	5137	5632	4.22	0.57
Calcium, mg/d	362	415	500	596	727	950	1025	3.41	0.79	350	380	458	562	704	771	854	5.00	0.76
Magnesium, mg/d	220	244	276	322	384	448	516	3.82	0.68	187	208	241	274	326	364	391	3.04	0.39
Iron, mg/d	6.4	7.2	8.0	9.5	11.0	13.0	14.3	3.84	0.65	5.6	6.0	7.4	8.3	9.4	11.5	12.6	3.13	0.50
Zinc, mg/d	6.5	7.2	8.2	9.3	10.7	12.6	13.9	3.31	0.63	5.6	6.1	7.0	8.0	8.9	10.2	10.7	3.45	0.55
Copper, mg/d	0.90	1.00	1.10	1.40	1.60	1.90	2.00	3.32	0.51	0.80	0.90	1.00	1.20	1.30	1.50	1.70	2.84	0.43
**Nutrients with DG**																		
Protein, % energy	12.8	13.4	14.3	15.2	16.4	17.6	18.1	2.81	0.09	13.9	14.2	14.9	15.7	16.8	17.8	18.7	3.94	0.57
Fat, % energy	20.0	20.5	23.1	25.8	29.1	33.1	34.4	3.29	0.57	21.6	22.6	25.3	27.3	29.5	31.9	32.3	3.17	-0.08
SFA, % energy	5.1	5.3	6.1	6.9	8.4	9.5	10.2	2.54	0.41	5.3	5.9	6.9	7.6	8.3	9.5	10.0	3.26	0.19
Carbohydrate, % energy	41.0	44.0	48.8	53.2	58.2	60.1	62.9	2.74	-0.43	48.7	50.3	52.8	56.2	59.2	62.2	63.2	3.28	-0.10
Dietary fibre, g/d	16.0	17.3	20.9	23.3	27.1	32.7	36.7	4.22	0.87	15.5	15.8	17.9	21.1	24.7	27.6	30.1	3.21	0.54
Sodium, g salt equivalent	8.8	9.1	10.1	11.3	12.9	14.7	15.8	9.40	1.75	6.8	7.1	8.8	10.0	11.8	13.0	14.3	4.22	0.57
Potassium, mg/d	1976	2187	2634	2999	3646	4424	5048	4.56	1.02	1777	2073	2334	2712	3319	3796	4094	2.89	0.39
**Nutrients with AI**																		
n-6 PUFA, g/d	5.80	7.30	9.10	10.70	12.80	15.50	18.10	4.77	0.97	6.40	6.70	7.60	8.90	10.30	11.60	12.20	2.65	0.35
n-3 PUFA, g/d	1.60	1.90	2.20	2.90	3.50	3.90	4.20	4.71	0.62	1.30	1.40	1.70	2.30	2.90	3.60	4.30	4.79	1.16
Vitamin D, μg/d	5.5	6.5	9.1	11.5	14.4	17.7	19.6	4.45	0.85	4.2	5.2	6.9	8.9	11.7	15.5	17.4	3.04	0.67
Vitamin E, mg/d	5.4	6.0	7.3	8.5	10.5	12.2	14.4	4.53	1.05	5.3	5.6	6.4	8.0	9.5	10.7	11.1	2.55	0.39
Vitamin K, μg/d	132	140	184	286	393	514	585	3.13	0.75	147	168	222	276	342	477	520	3.50	0.86
Pantothenic acid, mg/d	4.70	4.90	5.90	6.80	7.70	9.30	10.50	7.42	1.57	4.10	4.50	5.00	6.00	6.90	7.90	8.50	3.96	0.77
Potassium, mg/d	1976	2187	2634	2999	3646	4424	5048	4.56	1.02	1777	2073	2334	2712	3319	3796	4094	2.89	0.39
Phosphorus, mg/d	823	922	1053	1221	1393	1643	1840	4.12	0.75	718	778	902	1067	1203	1370	1410	3.45	0.30
Manganese, mg/d	2.80	3.30	3.80	4.60	5.40	7.10	7.80	12.68	2.28	2.30	2.60	3.20	3.90	4.90	5.30	6.00	2.98	0.38

AI, Adequate Intake; DG, Tentative Dietary Goal for Preventing Lifestyle-related Diseases; DRIs, Dietary Reference Intakes; EAR, Estimated Average Requirement; NE, niacin equivalent; SFA, saturated fatty acid; PUFA, polyunsaturated fatty acid; RAE, retinol active equivalent. [a] Usual intake was estimated using the Multiple Source Method based on 8-day weighed dietary-record data.
